# Photo-antagonism of the GABA_A_ receptor

**DOI:** 10.1038/ncomms5454

**Published:** 2014-07-29

**Authors:** Martin Mortensen, Favaad Iqbal, Arun P. Pandurangan, Saad Hannan, Rosemary Huckvale, Maya Topf, James R. Baker, Trevor G. Smart

**Affiliations:** 1 Department of Neuroscience, Physiology and Pharmacology, University College London, Gower Street, London WC1E 6BT, UK; 2 Department of Chemistry, University College London, 20 Gordon Street, London WC1H 0AJ, UK; 3 Institute of Structural and Molecular Biology, Crystallography/Department of Biological Sciences, Birkbeck College, University of London, London WC1E 7HX, UK

## Abstract

Neurotransmitter receptor trafficking is fundamentally important for synaptic transmission and neural network activity. GABA_A_ receptors and inhibitory synapses are vital components of brain function, yet much of our knowledge regarding receptor mobility and function at inhibitory synapses is derived indirectly from using recombinant receptors, antibody-tagged native receptors and pharmacological treatments. Here we describe the use of a set of research tools that can irreversibly bind to and affect the function of recombinant and neuronal GABA_A_ receptors following ultraviolet photoactivation. These compounds are based on the competitive antagonist gabazine and incorporate a variety of photoactive groups. By using site-directed mutagenesis and ligand-docking studies, they reveal new areas of the GABA binding site at the interface between receptor β and α subunits. These compounds enable the selected inactivation of native GABA_A_ receptor populations providing new insight into the function of inhibitory synapses and extrasynaptic receptors in controlling neuronal excitation.

## Abstract (web summary)

The GABA_A_ receptor has an important role in brain function. Here Mortenson *et al.* design and synthesize photoactivatable compounds based on the GABA_A_ antagonist gabazine and show that these can be used to inactivate the receptor, to identify new residues at the GABA binding site and can be adapted for trafficking studies.

## Graphical abstract

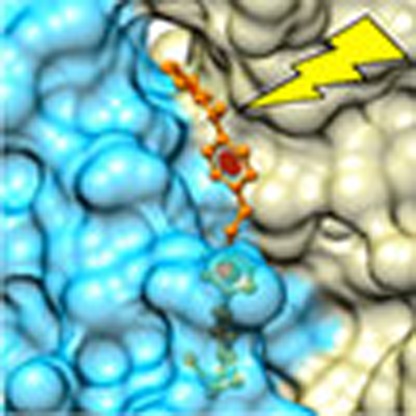

The precise coordination of our behaviour requires that we have adequate temporal control over neuronal excitation. The responsibility for this control falls largely to γ-aminobutyric acid type A receptors (GABA_A_Rs). The timing, extent and cellular location of synaptic inhibition have a critical impact on neural network activity and therefore behaviour^1^,^2^,^3^,^4^,^5^. Under normal circumstances, inhibition will be regulated by endogenous factors, post-translational modifications and by plasticity mechanisms. It is therefore unsurprising that dysfunction to GABAergic inhibition is implicated in numerous neurological diseases^6^,^7^,^8^.

The strength (or macroscopic efficacy) of synaptic inhibition will depend on many factors, not least the number of GABA_A_Rs clustered at the postsynaptic membrane, and the mean probability of GABA channel opening. Receptor clustering will be affected by numerous signalling pathways, including GABA_A_R phosphorylation^9^,^10^; while channel opening will be a function of the GABA concentration in the synaptic cleft and the activity of allosteric modulators, such as the neurosteroids^11^. Of equal importance for effective synaptic inhibition is the potential for different GABA_A_R isoforms with their attendant differences in physiological and pharmacological properties, to be targeted to specific domains (inhibitory synapses) in the same cell^12^,^13^.

To understand how this exquisite targeting of GABA_A_Rs to specific membrane domains in single cells relates to their impact on neural activity requires a method to modulate, irreversibly inactivate and/or to track the movement of such receptors. This can be partly achieved with fixed tissue by using receptor subtype-specific antibodies. Unfortunately this method will not allow any measure of real-time receptor dynamics^14^. By contrast, we can express GABA_A_R subunits that carry either mutations to critical structures (for example, ion channel)^15^, or are tagged with fluorophore labels^16^ to reveal real-time dynamics in live cells. The latter approaches, although extremely useful, nevertheless require the expression and monitoring of recombinant receptor protein expressed in native cells, and thus, the behaviour of native GABA_A_Rs can only be ascertained by inference.

Here we take a different approach to enable the direct study of native GABA_A_Rs. This requires the design of novel ligands that can be attached, and irreversibly bound when appropriately activated, to native GABA_A_Rs. Using available knowledge of the interfacial GABA binding sites on the GABA_A_R^17^, we have developed a class of ligands that can photoinactivate GABA_A_Rs. These ligands have two major advantages over prior methods: first, we can track native GABA_A_Rs *in situ* without the need for recombinant receptor expression in neurons, and second, by choosing a ligand that occludes the GABA binding site, we can specifically inactivate populations of GABA_A_Rs in particular areas thereby gaining valuable insight into their function and trafficking, in addition to revealing the importance of membrane delimited inhibition.

## Results

### Designing a photoactivated GABA_A_R antagonist

**Figure 1:**
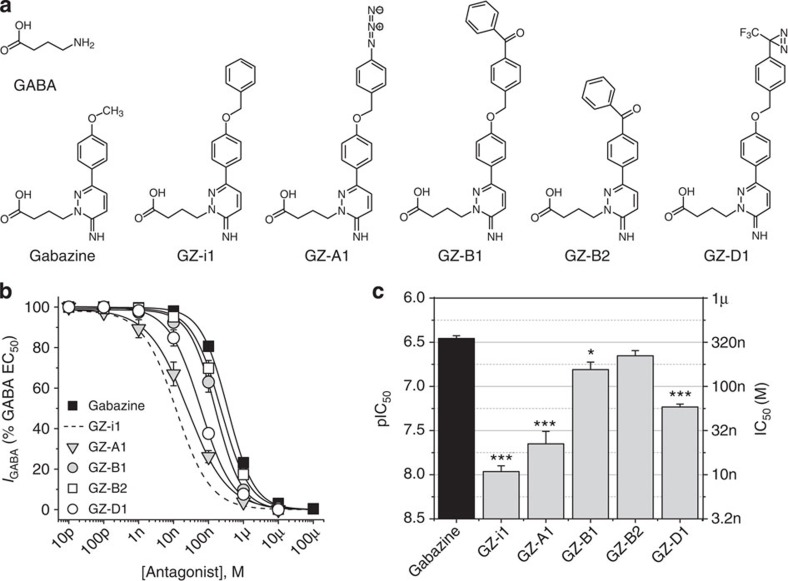
Photoactivated gabazine analogues are antagonists at α1β2γ2S GABA_A_ receptors. (**a**) Structures of GABA, gabazine and the new gabazine analogues: GZ-i1 (intermediate), GZ-A1 (azide), GZ-B1 and GZ-B2 (benzophenones) and GZ-D1 (diazirine). (**b**) GABA current inhibition curves for gabazine and gabazine analogues. The data are normalized (%) to the currents activated by an EC_50_ (~10 μM) for GABA (*n*=6–8 cells). Previous data for GZ-i1 is shown as a dotted line for comparison^27^. (**c**) Bar graph of antagonist potencies depicted as pIC_50_ (left ordinate) and IC_50_ (right; nM). The s.e. values only correspond to the pIC_50_ values. *P<0.05; ***P<0.001; *t*-test *n*=6–8). All data points and bars represent mean values±s.e.m.

We selected gabazine as the lead structure for synthesizing new photoactive reagents for several reasons: (i) It is a competitive GABA_A_R antagonist that binds to residues in the GABA recognition/binding site preventing agonist-dependent receptor activation. This strategy of causing just inhibition was preferred to photoactive allosteric modulators (often anaesthetics^18^,^19^), since these have multiple effects inducing inhibition and also concurrent activation and potentiation at GABA_A_ receptors; (ii) gabazine exhibits partial negative allosteric modulation by inhibiting GABA_A_R activation by pentobarbital (barbiturate) and alphaxalone (steroid) from their discrete binding sites on the receptor^20^; (iii) gabazine contains an easily identified ‘GABA structure’ in the molecule that is unencumbered by other groups, unlike a similar GABA moiety in bicuculline^21^, which is another competitive GABA_A_R antagonist^22^,^23^; and (iv), the phenoxy group on gabazine presents a chemically convenient site for attaching photoactivatable groups (Fig. 1a).

### Chemistry of gabazine analogues

To maximize the prospects of obtaining high potency gabazine analogues, we took note of several key structure–function characteristics of ligands that bind effectively to the GABA binding site. As the carboxy- and amino-ends of GABA are important for its engagement at the GABA binding site^24^, and the carboxyl side-chain of the GABA moiety in gabazine is crucial for antagonism^25^, we avoided making any modifications to these parts of the gabazine molecule. We also noted that the aromatic ring at position 6 on the pyridazine ring was important in affording gabazine its potency, and should therefore be retained^25^,^26^,^27^ (Fig. 1a). Thus, we chose to concentrate on the phenoxy group as the point of attachment for the photoactivatable groups, having shown in initial synthetic studies that the incorporation of a benzyl group led to a further increase in potency (GZ-i1, Fig. 1a)^27^.

The following three types of photoactive groups were incorporated into gabazine: an aryl azide^28^ (GZ-A1), a benzophenone^29^ (GZ-B1) and an aryldiazirine^30^ (GZ-D1; Supplementary Fig. 1a). A second truncated benzophenone–gabazine analogue was also synthesized, where the phenoxy ring of gabazine was directly replaced by the benzophenone (GZ-B2; Fig. 1a; Supplementary Fig. 1b). When these photoactive groups are exposed to ultraviolet (UV) light (wavelength ~300–370 nm) they respond by forming highly reactive intermediates. In the case of aryl azides and diazirines, this involves the loss of N_2_ to afford a nitrene or carbene, respectively, while the benzophenones form a photoexcited state that behaves as a diradical. In each case, the reactive species can then react and covalently attach to nearby amino-acid residues in the GABA binding site.

### Photoactive analogues are high potency inhibitors at GABA_A_Rs

**Figure 2:**
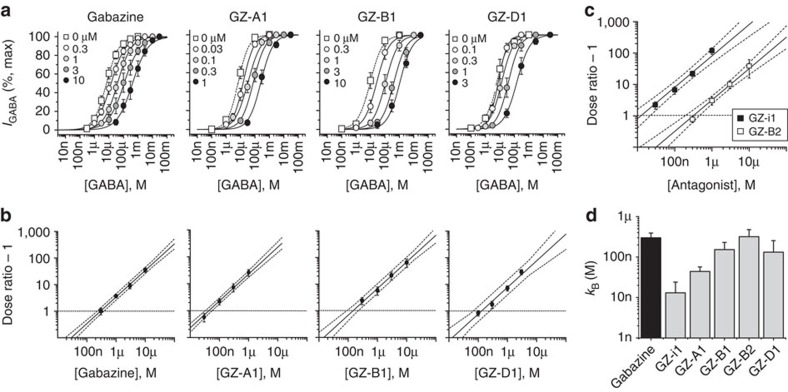
Affinities of photoactive gabazine analogues. (**a**) GABA concentration–response curves constructed for α1β2γ2 receptors in the absence and presence of increasing concentrations of the antagonists: gabazine, GZ-A1, GZ-B1 and GZ-D1 (*n*=5–7). (**b**) Schild analysis plots for gabazine and each analogue were derived from **a**. The linear regression lines are constrained to a slope of 1, indicative of competitive-type of antagonism. Confidence intervals (±95%) are shown as dotted lines. Horizontal dotted line intercept indicates the antagonist equilibrium constant, *k*_B_. (**c**) Schild plots for GZ-i1 and GZ-B2. (**d**) Bar graph of *k*_B_ values for gabazine and all analogues determined by the Schild analysis. All data points and bars represent mean values±s.e.m.

We first assessed the gabazine analogues for their potency in antagonizing a GABA EC_50_ response using the synaptic-type recombinant α1β2γ2 GABA_A_ receptor expressed in HEK cells. This would determine if the photoactive groups are accommodated by the GABA binding site. The synthetic compound, GZ-i1, is an intermediate between gabazine and its photoactive analogues. The simple addition of a phenyl ring increased the potency of gabazine by more than 30-fold^27^ (Fig. 1b,c), in accord with the 20-fold increase in affinity (K_i_) of GZ-i1 measured using Schild analysis (Fig. 2c,d).

Surprisingly, the relative potencies of the photoactive compounds, GZ-A1 (azide), GZ-B1 (benzophenone) and GZ-D1 (diazirine), were 1.5- to 30-fold higher than that of gabazine, with the exception of the truncated benzophenone, GZ-B2, which was equipotent (Fig. 1b,c). While these potency comparisons are dependent on the GABA concentration used, the affinities of the photoactive gabazine analogues are not as they are determined directly using a Schild analysis for competitive antagonism^31^ (Fig. 2). The antagonist dissociation constants (*k*_B_, nM) decreased in the order: GZ-B2 (318)>Gabazine (300)>GZ-B1 (153)>GZ-D1 (132)>GZ-A1 (44)>GZ-i1 (13); Fig. 2d). Such a rank order was unexpected if the molecular volume of the photoactive side-chain was the major limiting factor for ligand binding. Thus, we concluded that these large photoactive groups in the phenoxy position of gabazine are fully accommodated at the GABA binding site. The increased affinity (lower *k*_B_) of the analogues must therefore result from increased interactions between gabazine analogues and binding site residues either via H-bonds, cation–π interactions, or π–π stacking of aromatic rings.

### Photoinactivation of recombinant GABA_A_ receptors

**Figure 3:**
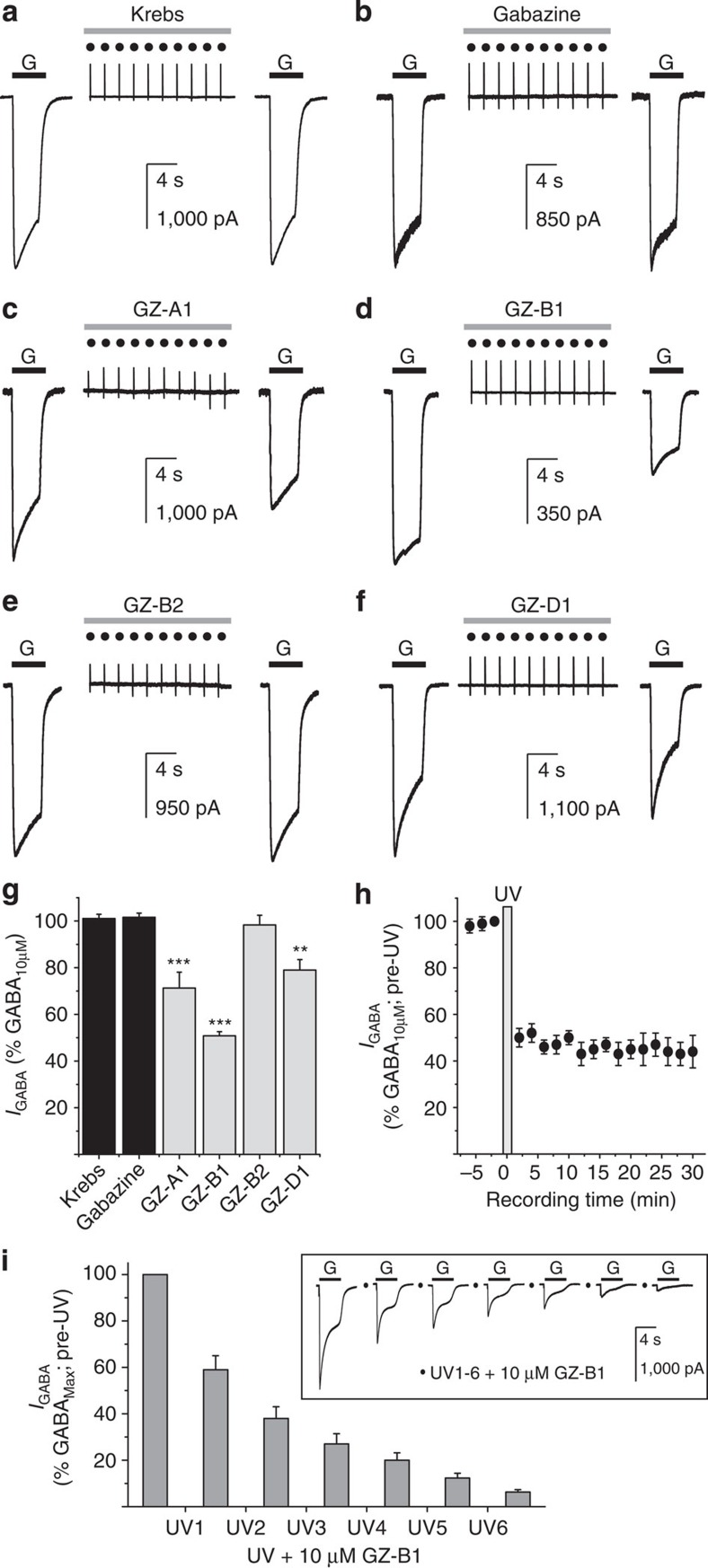
Irreversible antagonism of GABA currents by photoactive gabazine analogues. Membrane currents activated by 10 μM GABA (G, black bar) on α1β2γ2 GABA_A_ receptors before and after a cycle of 10 brief UV flashes (dots) under control conditions (Krebs, **a**) and following exposure (grey bar) to 10 μM: gabazine (**b**), GZ-A1 (**c**), GZ-B1 (**d**), GZ-B2 (**e**) or GZ-D1 (**f**). A 2-min interval was inserted between the first GABA application and the UV exposure protocol, while 3–5 min separated the UV protocol from the second GABA application. This latter interval was sufficient to ensure complete dissociation of all antagonists that were not covalently bound to the receptor. (**g**) Bar graph of irreversible inhibition caused by gabazine and photoactivated gabazine analogues of 10 μM GABA currents, normalized to control currents in Krebs (=100%). **P<0.01; ***P<0.001 for comparison with gabazine (*n*=7–12; *t*-test). (**h**) Time course of GZ-B1 irreversible inhibition of responses to 10 μM GABA. UV exposure indicated by the dots. (**i**) Bar graph showing increased current inhibition with successive cycles of UV exposure (*n*=5); inset: typical GABA currents before and after cycles of UV exposure. All data points and bars represent mean values±s.e.m.

The photoactive capabilities of the azide, benzophenone and diazirine groups on the gabazine molecule to covalently link to the GABA binding site were studied using whole-cell recording from HEK cells expressing α1β2γ2S GABA_A_ receptors. The gabazine analogues, GZ-A1, GZ-B1, GZ-B2 and GZ-D1, were selected, in conjunction with a photoactivation protocol involving UV exposure. The intensity and duration of exposure were titrated to ensure photoactivation of the compounds without perturbing cell health, ascertained by measuring the membrane leak current and access resistance. Control whole-cell GABA-activated currents, recorded before and after applying the photoactivation protocol (see Methods) in the presence of Krebs alone were unchanged (101.1±1.8%; mean±sem; *n*=7; Fig. 3a). This verified that under our conditions, UV light exposure did not damage cells or change GABA potency for α1β2γ2 receptors^32^. Similarly, no reduction in the GABA-induced current was observed after applying the photoactivation protocol with gabazine (10 μM; 101.6±3.3%; *n*=7), indicating that the parent molecule has no innate photoreactivity, and that 3–5 min is sufficient, after UV exposure, for the antagonist to dissociate from the GABA binding site (Fig. 3b).

For the azide-linked gabazine analogue, GZ-A1, the GABA-induced current was reduced irreversibly post-UV by ~30% (to 71.3±6.8%; *n*=7; Fig. 3c,g). For the two benzophenone-linked gabazine analogues, the post-UV GABA current was irreversibly reduced by GZ-B1 (to 50.8±1.8%; *n*=12; Fig. 3d,g), but not by the truncated version, GZ-B2, lacking one phenyl ring (98.3±4.2%; *n*=7; Fig. 3e,g). In comparison, the diazirine-linked analogue, GZ-D1, irreversibly reduced GABA current by ~20% (to 79.0±4.5%; *n*=7; Fig. 3f,g). The most efficacious molecule inducing irreversible block at the GABA binding site was therefore the ‘extended’ benzophenone–gabazine analogue, GZ-B1, which was selected for further characterization. The irreversible nature of the inhibition was evident from extended recording periods of at least 30 min post-UV exposure (Fig. 3h). The unchanging extent of inhibition and lack of recovery also re-affirmed that surface GABA_A_ receptors in HEK cells are not replaced during this period^15^. Ablation of the agonist response was routinely achieved with successive cycles of UV exposure in the presence of 10 μM GZ-B1 (Fig. 3i). To ensure that some agonist response remained for the measurement of potencies, we used a single UV exposure cycle in the presence of GZ-B1.

### GZ-B1 has lower potency at α3β3γ2 and α4β3δ GABA_A_ receptors

**Figure 4:**
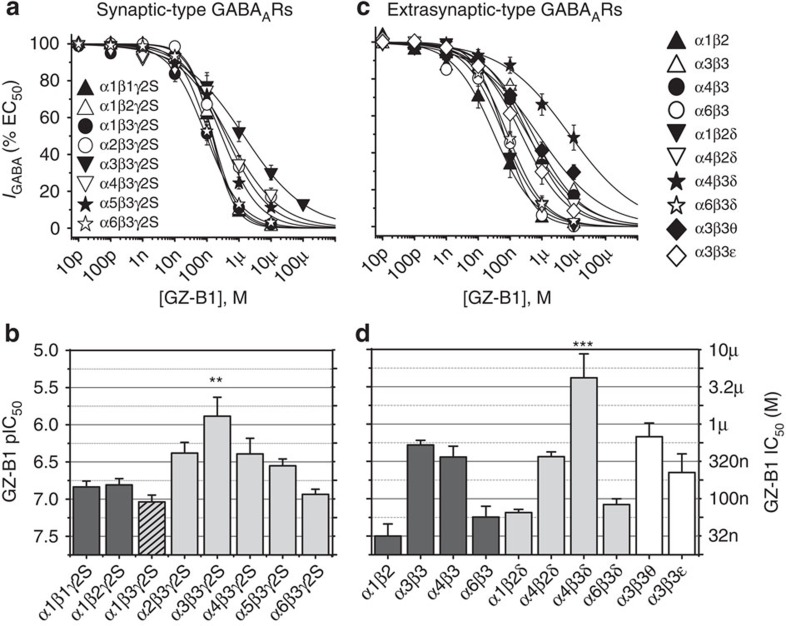
Inhibition by GZ-B1 at synaptic- and extrasynaptic-type GABA_A_ receptors. Inhibition curves for GZ-B1 of GABA EC_50_ responses on synaptic- (**a**) and extrasynaptic-type (**c**) receptors with corresponding bar graphs (synaptic (**b**), extrasynaptic (**d**)). Potency is plotted as pIC_50_ (left axis) and as IC_50_ (μM, right axis) in each bar graph (*n*=5–9). **P<0.01; ***P<0.001 follows a comparison of pIC_50_ values for all GABA_A_ receptor isoforms with α1β3γ2 (hatched; ANOVA). All data points and bars represent mean values±s.e.m.

To determine if GZ-B1 exhibited receptor subtype selectivity, we examined its inhibitory profile for 18 synaptic- and extrasynaptic-type GABA_A_ receptors, selected because they are likely to be expressed in the central nervous system^33^,^34^. By varying the highly homologous β-subunits (β1–3) in synaptic-type α1βxγ2 receptors, GZ-B1 potency (IC_50_) remained constant (analysis of variance (ANOVA); *P*=0.26; Fig. 4a,b). Conducting a similar examination with different α subunits in α1-6β3γ2 receptors, GZ-B1 potency was significantly reduced at α3β3γ2 compared with either α1β3γ2 (*P*<0.001, ANOVA with Tukey–Kramer *post hoc* tests) or α6β3γ2 (*P*<0.01; Fig. 4a,b). For the prospective extrasynaptic-type receptors, GZ-B1 potency significantly varied in the αβ and αβδ subgroups (ANOVA, *P*<0.0001), being higher at α6β3 compared with α3β3 (*P*<0.001) and α4β3 receptors (*P*<0.01; Fig. 4c,d), and also higher at α6β3δ compared with α4β3δ receptors. Potency was unaffected by including the δ-subunit with α1β2 or α6β3 receptors, but was reduced by its inclusion in α4β3 receptors (*P*<0.05). By comparison, potency was unaltered by incorporating either θ or ε subunits into α3β3 receptors (Fig. 4c,d). Comparing the selected synaptic and extrasynaptic GABA_A_ receptors with α1β3γ2 revealed significantly lower potencies for GZ-B1 at α3β3γ2 and α4β3δ receptors (ANOVA, Dunnett *post hoc* test, Fig. 4b,d).

### Ligand docking using a GABA_A_ receptor model based on AChBP

**Figure 5:**
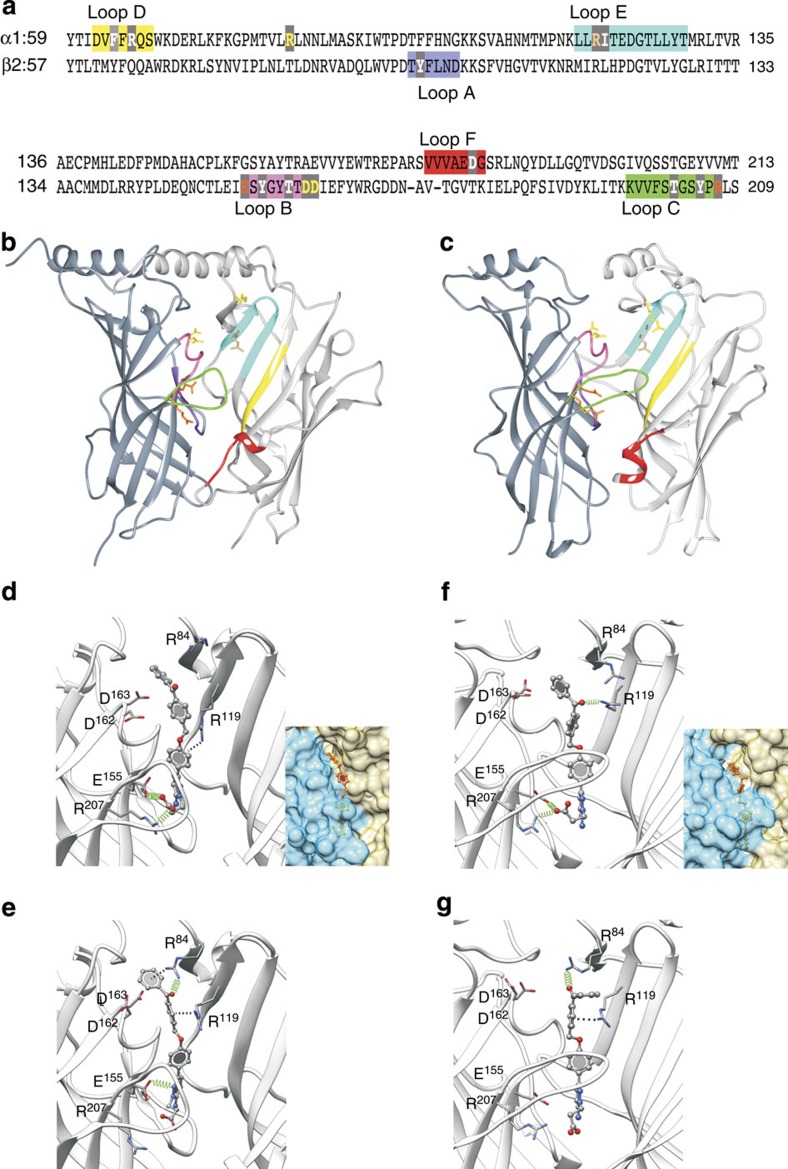
GABA binding site model with docked GZ-B1 molecule. (**a**) Primary sequence alignment of murine GABA_A_ receptor α1 and β2 subunits. Binding loops A–C on β2 and D–F on α1 are colour coded. Key residues (grey boxes) involved in reversible binding of GZ-B1 are shown in orange (β2-E155, β2-R207), residues involved in irreversible binding to photoactivated GZ-B1 are shown in yellow (α1-R84, β2-D162, β2-D163), and a residue involved in both reversible and irreversible binding is shown in beige (α1-R119). Other residues important for GABA binding are shown in white. (**b**) Model of the GABA binding site between β2 (dark grey) and α1 (light grey) subunits based on AChBP (ribbon form). (**c**) Model of the GABA binding site between β2 (dark grey) and α1 (light grey) subunits based on GluCl (ribbon form). Loops A–F, α1-R84, 119 and β2-E155, D162, 163 and R207 on **b**,**c** are shown using colour code as in **a**. (**d**,**e**) Predicted binding modes for GZ-B1 based on AChBP using unconstrained (**d**) and scaffold-match-constrained (**e**) docking. (**f**,**g**) Predicted binding modes for GZ-B1 based on GluCl using unconstrained (**f**) and scaffold-match-constrained (**g**) docking. (**d**,**f** insets) Subunit interface surfaces (β2 is blue; α1 is green) are shown with the benzophenone of GZ-B1 protruding from underneath loop C and settling in a cavity above the GABA binding site. The unconstrained binding modes for GZ-B1 (**d**,**f**) predicted an interaction with R207 (β2), E155 (β2) and R119 (α1). For AChBP, the scaffold-match-constrained binding mode for GZ-B1 (**e**) predicted H-bond formation with R84 (α1) and E155 (β2) and a cation–π interaction with R119 (α1). For GluCl, the scaffold-match-constrained binding mode for GZ-B1 (**g**) predicted H-bond formation with R84 (α1) and a cation–π interaction with R119 (α1). The H-bonds are shown as spring representation. Cation–π interactions are depicted as dashed black lines.

To understand how GZ-B1 binds within the GABA site, we first performed GOLD^35^ docking simulations of GABA, gabazine and GZ-B1 with the α1β2γ2 GABA_A_ receptor modelled on the 2 Å resolution crystal structure of the unliganded acetylcholine binding protein (apo-AChBP, PDB ID: 2BYN). This template was initially selected because loop C, which caps the binding site when occupied by an agonist^36^,^37^, is uncapped, but not overtly displaced outwards, as observed when a large competitive antagonist is bound to the same site^36^. For antagonists of comparable size to gabazine and GZ-B1, such as methyllycaconitine, the positioning of loop C in AChBP is unchanged (PDB: 2BYR)^36^. The GABA binding site is located at β–α subunit interfaces surrounded by residues from six binding loops designated as: A, B, and C from the ‘+’ face of the β subunit and D, E and F from the ‘−’ face of the α subunit^37^,^38^ (Fig. 5a,b). From all the docking results, the most probable binding mode was selected based on its ranking, its similarity to GABA interactions with the GABA_A_R as reported in the literature and the frequency of its similarity to the other binding modes in the diverse docking solutions.

Docking GABA, gabazine or GZ-B1 into the GABA site identified several charged residues potentially involved in binding (Fig. 5a,b). Some of these have been previously implicated in GABA binding^39^. By docking GABA, we identified two solutions (ranked 1 and 2) that predict two different binding modes whereby the carboxyl group of GABA formed H-bonds with R119 (α1, rank 1) or E155 (β2) and R207 (β2, rank 2) (Supplementary Fig. 2a). In addition, for the rank 1 solution, H-bonds are also formed with S156 (β2), G158 (β2), Y159 (β2) and Y205 (β2), and for the second ranked solution, H-bonds are formed with Y97 (β2) and a cation–π interaction with Y157 (β2). The interacting residues are spatially spread around the GABA binding site and hence we predict that GABA potentially binds to the receptor in two modes. Such interactions have been previously shown to be involved in GABA binding^40^,^41^.

From the gabazine docking, we examined the top 2 ranked solutions (rank 1 and 2). Rank 1 only had one H-bond interaction between the carboxyl group of gabazine and R119 (α1). However for rank 2 the key carboxyl group formed H-bonds with the receptor residues, R207 (β2) and E155 (β2), and the aromatic ring was also engaged in a cation-π interaction with R119 (α1) (Supplementary Fig. 2b). These interactions were also evident with the top 2 solutions for GABA docking elevating rank 2 as a potential binding mode compared with the other docking solutions. In addition, based on the root mean squared deviation (r.m.s.d.) measure, rank 2 was found to be part of a cluster of similar binding modes. The cluster contained 24% (12/50) of the diverse docking solutions, including ranks 3 and 4 (Supplementary Fig. 2c).

For the docking of GZ-B1, we applied a two-stage docking protocol (Methods). A potential binding mode (Fig. 5d) was first identified based on the observation that GZ-B1 was interacting with similar residues (R207 (β2), E155 (β2) and R119 (α1)) to those identified in the GABA docking study. Moreover, we expected GZ-B1 to interact similarly to gabazine, given that GZ-B1 and gabazine share a core structure. Based on the r.m.s.d. measure, the observed binding mode was similar in 28% (14/50) of the diverse docking solutions, including ranks 3 and 5. (Supplementary Fig. 2d). Next, we explored the binding mode of GZ-B1 using constraint docking by positioning GZ-B1 in the binding site enabling residues that could covalently bind to the photoactivated benzophenone group to be identified (Methods). With ‘scaffold-match’ constraints, the activated oxygen of the benzophenone group was consistently predicted to form an H-bond with R84 (α1) in our top 3 ranked solutions (rank 1, Fig. 5e). This ‘region-constraint’ docking method also identified interactions with either D162 (β2) and/or D163 (β2) (data not shown).

### Ligand docking using a GABA_A_ receptor model based on GluCl

The predicted binding mode for GZ-B1 obtained from the first stage of docking involved H-bonding with R207 (β2), E155 (β2) and R119 (α1) (Fig. 5f). This binding mode was similar in 32% (16/50) of the diverse docking solutions, including ranks 2, 3 and 4, representing the most populated binding mode (Supplementary Fig. 2e). Intriguingly, the two-stage docking protocol predicted a similar binding mode to that observed using the AChBP template and the scaffold-match constraint. This identified an H-bond between the activated oxygen of the benzophenone group and R84 (α1) (rank 1, Fig. 5g). However, interactions with D162 (β2) and D163 (β2) were not predicted to occur either from two-stage docking or from region-constraint docking.

The docking results predicted that GABA and gabazine are bound completely within the GABA site behind loop C, whereas the benzophenone group of GZ-B1 projects up along the β–α subunit interface and out from under loop C, before re-entering the interface and terminating near a new cavity between β and α subunits (Fig. 5d,f). This cavity is predicted to penetrate through to the external vestibule located above the ion channel. The intersubunit space around the cavity is considered unimportant for GABA activation of the receptor, but its volume is such that competitive antagonists with additional moieties can be accommodated without impeding binding. Another interesting observation is that among the unconstrained docking results, the aromatic ring of GZ-B1 was always orientated towards the extracellular domain in 68 and 84% of the solutions based on AChBP and GluCl, respectively. This preferred orientation of GZ-B1 within the GABA binding site is also supported by the proposed binding mode (Fig. 5d,f).

### Mutating the binding site for GABA, gabazine and GZ-B1

**Figure 6:**
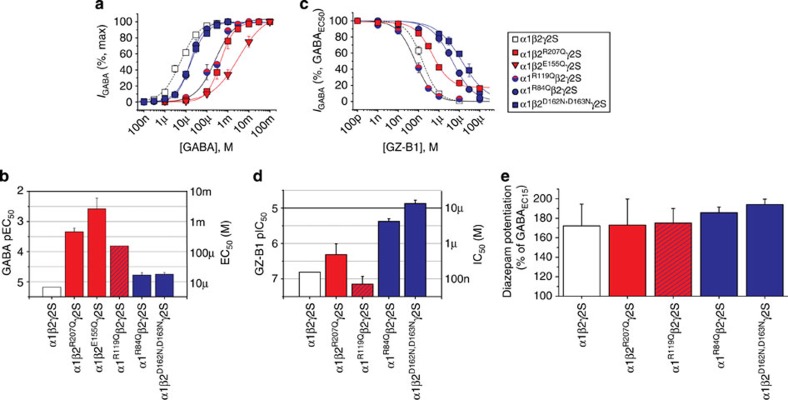
Binding site residues affecting GABA and GZ-B1 potencies. (**a**) GABA concentration–response curves for wild-type and mutated α1β2γ2 receptors containing: β2^R207Q^, β2^E155Q^, α1^R119Q^, α1^R84Q^ or β2^D162N, D163N^. (**b**) Bar graph of GABA pEC_50_s (left) and EC_50_s (right). (**c**) Inhibition curves for GZ-B1 (inhibiting the GABA EC_50_) on wild-type and mutant receptors as in *A*, except α1β2^E155Q^γ2S. The key applies to **a**,**c**. (**d**) Bar graph of GZ-B1 pIC_50_s (left) and IC_50_s (right; *n*=5–6). White symbols represent wild-type data; red reflects mutated GABA binding site residues (β2^R207Q^, β2^E155Q^); blue/red shows data for R119Q that can affect GABA binding and/or GZ-B1; while blue represents residues involved in binding to the UV-activated oxygen of benzophenone (α1^R84Q^, β2^D162N+D163N^). (**e**) Diazepam (10 μM) potentiation of GABA EC_15_ responses for wild-type and β2^R207Q^, α1^R119Q^, α1^R84Q^ or β2^D162N, D163N^-mutant α1β2γ2 receptors (*n*=4–8; ANOVA *P*=0.84). All data points and bars represent mean values±s.e.m.

To examine the predictions from docking simulations that R119 (α1), E155 (β2) and R207 (β2) bind GABA, gabazine and GZ-B1, we replaced them with similar-sized uncharged glutamines. Substituting R119 (α1^R119Q^β2γ2) substantially reduced GABA potency (EC_50_: 155 μM), while gabazine (IC_50_: 188 nM) and GZ-B1 (IC_50_: 72 nM) potencies were increased by ~2-fold, compared with wild type (Fig. 6a–d; Supplementary Table 1).

Exchanging R207 (α1β2^R207Q^γ2) reduced the potencies for GABA (EC_50_: 452 μM), gabazine (IC_50_: 1.71 μM), and GZ-B1 (IC_50_: 487 nM; Fig. 6a–d; Supplementary Table 1), consistent with its strong role in the binding of GABA and the competitive antagonists. For E155Q (α1β2^E155Q^γ2S), a substantial leak current was evident in the absence of GABA (Supplementary Table 1) reflecting spontaneously open receptors (P~0.7). The small GABA-induced currents (<100 pA) indicated GABA potency was ~400-fold lower (EC_50_: 2.6 mM) than at wild-type receptors (Supplementary Table 1). Spontaneous channel opening^42^ made conventional assessment of antagonist potency difficult as the maximum GABA currents were reduced as expected. Therefore, we examined the inhibition of spontaneous channel activity by gabazine and GZ-B1 (relying on their negative allosteric properties), which revealed very low potencies (IC_50_s: >100 μM; Supplementary Table 1). Thus, as predicted following previous studies^39^,^40^,^42^,^43^,^44^,^45^,^46^,^47^, these residues are likely to affect the binding of the three ligands with potential effects, exemplified by E155Q, on channel gating.

### Residues outside the GABA binding site interact with GZ-B1

The three charged residues, R84 (α1) and D162/D163 (β2), identified as potential binding residues for the benzophenone group of GZ-B1, were replaced by either glutamine (R84Q) or asparagine (D162N, D163N). GABA potency was minimally affected by α1^R84Q^β2γ2 (EC_50_:17 μM) and α1β2^D162, D163N^γ2 (EC_50_:17 μM; Fig. 6a,b; Supplementary Table 1), as expected, due to their remote location from the GABA binding site. However, α1^R84Q^ and β2^D162N,D163N^ significantly reduced the potency of GZ-B1 (Fig. 6c,d; Supplementary Table 1), suggesting potential importance for binding the benzophenone group.

The double mutant, α1^R84Q^β2^R207Q^γ2, which includes the two key residues proposed to anchor each end of the GZ-B1 molecule in the binding site, reduced GZ-B1 potency by a 1,000-fold (IC_50_: 182 μM), while only halving GABA potency compared with β2^R207Q^ alone (452 μM to 955 μM; Supplementary Table 1).

The impact of the β2^E155Q^ mutation on ligand binding is difficult to interpret as it clearly affects the ability of the ion channel to remain shut in the absence of agonist. To verify that the other mutations are only locally affecting the GABA binding site and not introducing major conformational perturbations into the receptor, we examined allosteric modulation of the GABA_A_ receptor. Specifically, benzodiazepine-induced modulation was unaffected (Fig. 6e).

### Photoactivated GZ-B1 irreversibly binds to α1-R84

The importance of α1-R84, β2-D162 and β2-D163 for irreversible binding following photoactivation of GZ-B1 was investigated using near-saturating concentrations of GZ-B1 before and after UV. We also examined α1-R119 as a likely candidate to engage in irreversible bond formation given its close proximity to the photoactivated oxygen of the benzophenone group in GZ-B1.

**Figure 7:**
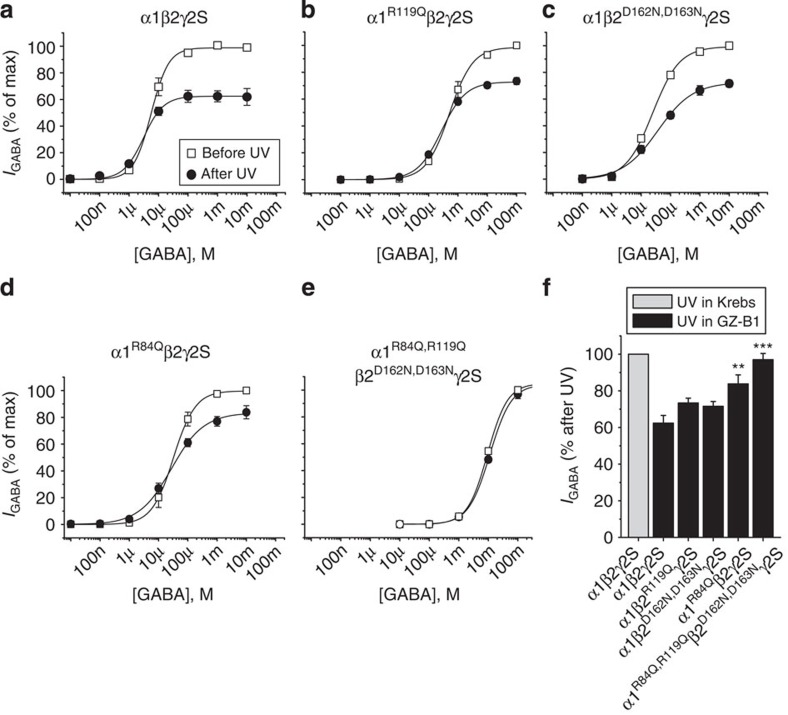
Binding site mutations reduce irreversible inhibition by GZ-B1. GABA concentration–response curves before and after photoactivation of either 10 μM GZ-B1 on: (**a**) α1β2γ2 and (**b**) α1β2^R119Q^γ2; or 100 μM GZ-B1 on: (**c**) α1β2^D162+163N^γ2; (**d**) α1^R84Q^β2γ2 and (**e**) α1^R84+119Q^β2^D162N, D163N^γ2 receptors (*n*=4–6). (**f**) Bar graph of maximum GABA currents after UV protocol in the presence of GZ-B1 for wild-type and mutated receptors. Currents are normalized to the maximum GABA current in Krebs (=100%). **P<0.01; ***P<0.001 compared with wild-type α1β2γ2 receptors in GZ-B after UV exposure (*t*-test). All data points and bars represent mean values±s.e.m.

The UV photoactivation protocol did not significantly affect GABA potency or macroscopic efficacy at wild-type receptors (α1β2γ2) in Krebs alone (Supplementary Table 2). For the wild-type α1β2γ2 receptor exposed to UV in the presence of GZ-B1, the maximum GABA current was reduced to 62±4.2% of control (*n*=6) due to irreversible block at the GABA binding site (Fig. 7a,f). The mutants, α1^R119Q^β2γ2 and α1β2^D162N,D163N^γ2, caused only a small or no reduction in the irreversible block of GZ-B1 when compared with wild-type (to 73±2.6%; *n*=6; *t*-test, *P*=0.05; and 71±2.6%; *n*=4; *t*-test, *P*=0.1491; respectively; Fig. 7b,c,f). However, α1^R84Q^β2γ2 caused a substantive reduction in the level of irreversible block (from 62% to only 84±4.9%; *n*=6; *t*-test, *P*=0.0067) indicating that α1-R84 is an important residue for binding of the photoactivated GZ-B1 molecule (Fig. 7d,f). Finally, we expressed a combined mutant, α1^R84,119Q^β2^D162,163N^γ2, which eliminated the GZ-B1 block (97±3.4%; *n*=4; *t*-test, *P*=0.0003; Fig. 7e,f). Thus, while α1-R84 is the most important binding partner for the photoactivated benzophenone group, α1-R119, β2-D162 and β2-D163 residues can, to a limited extent, affect the covalent binding of photoactivated GZ-B1 molecules.

### Photoactivated GZ-B1 irreversibly reduces synaptic inhibition

**Figure 8:**
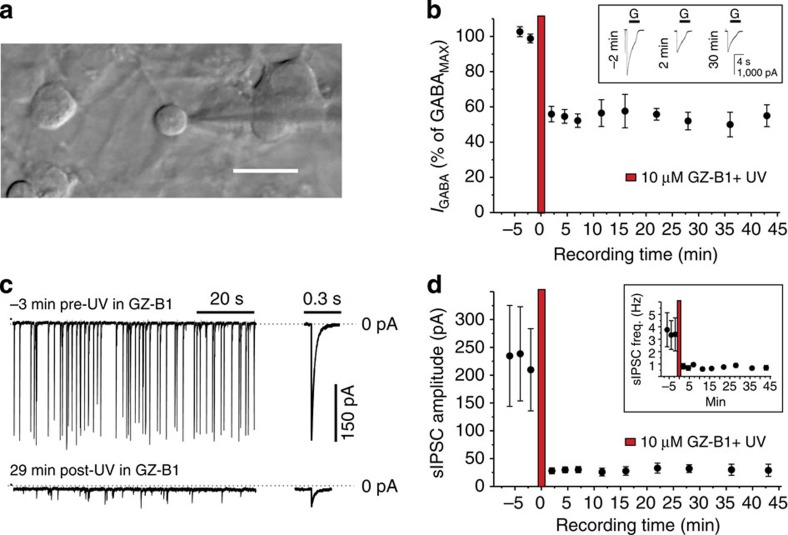
Inhibition of sIPSCs in cerebellar granule neurons by photoactivated GZ-B1. (**a**) Patch-clamped cerebellar granule neuron at 10DIV. Scale bar, 20 μm. (**b**) Time profile for 1 mM GABA-activated currents before and after 10 μM GZ-B1 and UV exposure (red bar). Inset: Examples of 1 mM GABA currents (G) 2 min before (−2 min), and 2 and 30 min after UV/GZ-B1. (**c**) sIPSCs from a single cerebellar granule neuron 3 min before and 29 min after UV/GZ-B1. Examples of individual sIPSCs are shown at a higher time resolution (right). (**d**) Time profile for sIPSCs after UV exposure with 10 μM GZ-B1 (red bar). Note the lack of recovery in **b**,**d** over 45 min. Inset: Graph showing that sIPSC frequency is also reduced after UV/GZ-B1.

To assess the ability of photoactivated GZ-B1 to reduce synaptic inhibition, we recorded from cultured cerebellar granule cells and monitored whole-cell GABA currents and spontaneous inhibitory postsynaptic currents (sIPSCs; Fig. 8a). Responses to rapidly applied GABA (1 mM) were depressed to a similar degree, after a single UV exposure, to those observed for recombinant α1β2γ2 GABA_A_ receptors. No recoveries were observed over 40–45 min following GZ-B1 photoactivation (Fig. 8b). Monitoring sIPSCs before and after an identical UV cycle in the presence of 10 μM GZ-B1 (Fig. 8c) revealed up to a 90% reduction in synaptic current amplitude, which did not recover during the recording (~45 min; Fig. 8d). This level of inhibition indicates that the synaptic receptors are highly sensitive to inhibition by photoactivated GZ-B1. The lack of recovery (both whole-cell GABA currents and sIPSCs) suggests that membrane insertion of GABA_A_ receptors from intracellular stores must be relatively slow.

### Tracking photolabelled GABA_A_ receptors

**Figure 9:**
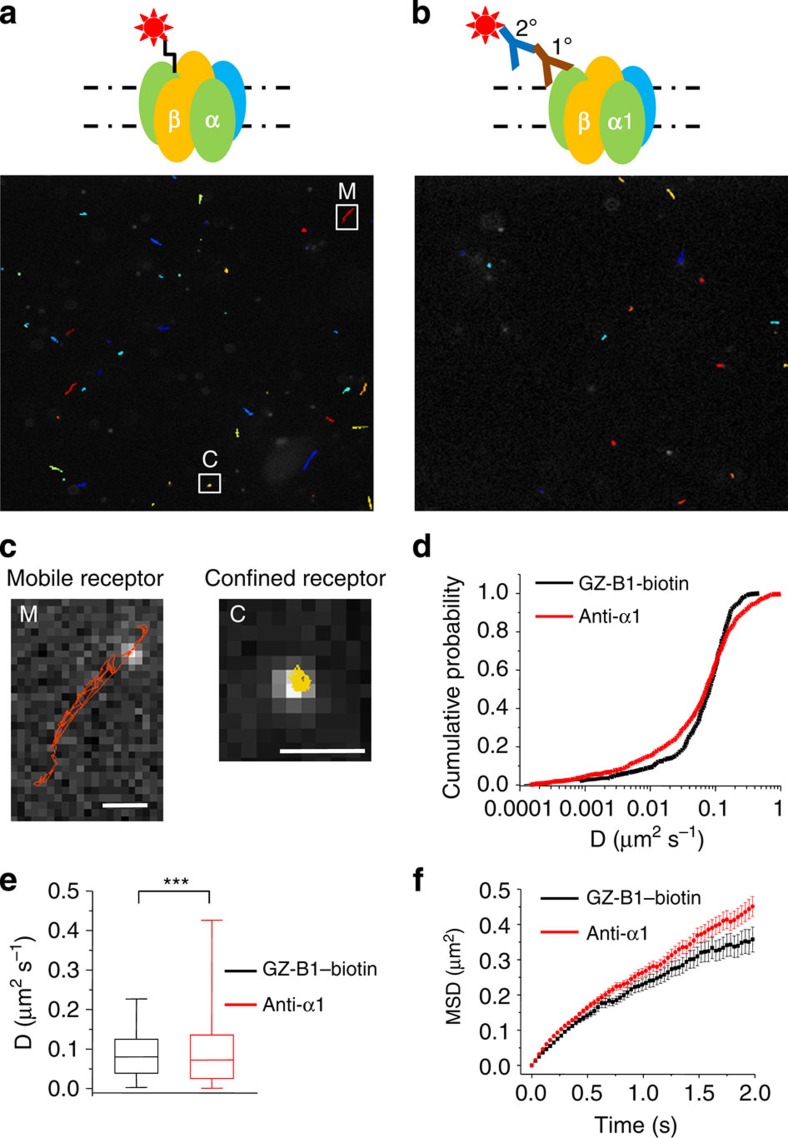
Mobility of QD-labelled GABA_A_ receptors on hippocampal neurons. (**a**,**b**) Schematics and trajectories for individual QDs photo-linked to GABA_A_ receptors via GZ-B1–biotin (**a**) and for GABA_A_ α1 subunits tagged with QDs via a primary antibody against α1 and a secondary antibody containing biotin and QD_655_–streptavidin (**b**). (**c**) Examples of trajectories from **a** of mobile (M) and confined (C) QDs/receptors. Confined QDs/receptors are most likely anchored at inhibitory synapses, whereas the more mobile QDs/receptors are thought to reside in the extrasynaptic domains. Trajectories were analysed using the ImageJ plug-in, SpotTracker 2D/3D and MatLab. Scale bars, 1 μm. (**d**) Diffusion coefficients of GABA_A_ receptors labelled with GZ-B1–biotin or with antibodies against α1 subunits. (**e**) Distribution of diffusion coefficients shown as a box-and-whisker plot (median, 25–75% interquartile range, whisker=5–95%) for GABA_A_ receptors tagged with GZ-B1 (*n*=446) or with anti-α1 antibodies (*n*=788; ***P<0.001, Kolmogorov–Smirnov test). (**f**) Mean square displacement (MSD) versus time plot of GABA_A_ receptors labelled with GZ-B1 and anti-α1 antibody. There was no significant difference in the confinement of the receptors. All data points and bars represent mean values±s.e.m.

The specific and irreversible binding of GZ-B1 to neuronal GABA_A_ receptors provided a means to label such receptors with fluorophores. We exploited this using a variation of GZ-B1 incorporating a polyethylene glycol linker attached to biotin (Supplementary Fig. 3a) designed to not interfere with photoactivation of GZ-B1 and its binding to GABA_A_ receptors. This moiety readily reacts with streptavidin-coated highly fluorescent quantum dots (QD_655_; Fig. 9a). By subsequently exposing these molecules to UV light, we labelled and then tracked the surface mobility of irreversibly inactivated GABA_A_ receptors on hippocampal neurons (Supplementary Fig. 3b,c,d; Fig. 9).

GABA_A_ receptors labelled with GZ-B1 exhibited both confined and mobile trafficking profiles in hippocampal neurons as expected for receptors that are confined at inhibitory synapses and for those that reside in the extrasynaptic domain (Fig. 9c). For comparison with GZ-B1, we also labelled separate GABA_A_ receptors with QDs on α1 subunits via a primary antibody to an external epitope (Fig. 9b). By tracking receptor mobility labelled with GZ-B1 or anti-α1 antibody, we determined the diffusion coefficients (*D*; Fig. 9d). The median *D* value after tracking individual QDs for anti-α1-labelled receptors (0.08; *n*=788) (Fig. 9e) was significantly reduced for GZ-B1–biotin-labelled receptors (0.07; Kolmogorov–Smirnov test, *P*<0.001; *n*=446 QDs). This probably reflects α1 subunit-containing GABA_A_ receptors predominantly located at synapses, which have lower *D* values, compared with GZ-B1–biotin-tagged receptors, which will include synaptic as well as the faster moving extrasynaptic GABA_A_ receptor populations. The mean square displacement plots for GABA_A_ receptors labelled with GZ-B1 (black) and anti-α1 antibody, revealed no significant difference in the confinement of the receptors. This is likely, as the ensemble of diffusion coefficients will include a mixed population of various synaptic and extrasynaptic receptors.

The utility of the GZ-B1–QD label is also emphasized in studying receptor internalization. Transfected hippocampal neurons expressing enhanced green fluorescent protein were labelled with GZ-B1–biotin–streptavidin–QD_655_ and incubated at 37 °C from 0 up to 60 min before fixation (Supplementary Fig. 4). Under these conditions, we followed the trafficking itineraries of receptors as they internalized into subcellular compartments (Supplementary Fig. 4; Supplementary Movie 1). Overall, the GZ-B1–QD complex forms a very useful label for tracking GABA_A_ receptor movement.

## Discussion

Dynamically regulating the number of GABA_A_ receptors at inhibitory synapses is a vital component of synaptic plasticity with implications for the long-term control of neuronal excitability, and for dysfunctional inhibitory transmission during neuropathological states. Monitoring the trafficking of synaptic receptors often requires antibodies recognizing an innate epitope, or a modified receptor structure to incorporate an epitope that is either recognized by selective antisera^48^, or is an inherent fluorophore^49^. Further modifications can enable the receptor to be coupled to a quantum dot^50^,^51^ or carry a mutation that is recognized by another ligand^15^. Although useful, such methods cannot be easily adapted to study native receptors. To address this problem, we devised a method that irreversibly inactivates native GABA_A_ receptors, using a new class of photoactivated GABA_A_ receptor antagonists. These can be used to investigate inhibition in various membrane domains and by linking the photoactivated antagonists to fluorophores, we can simultaneously investigate both receptor function and receptor trafficking.

Gabazine is an ideal lead compound due to its high affinity for the GABA binding site, its suitability for chemical synthesis, and the ease by which structural modifications can be made^25^,^26^. By attaching photoreactive groups to the phenoxy-end of gabazine, away from the GABA backbone, we found that these analogues retained or even increased their affinity for the GABA binding site. This feature was also noted by attaching a benzyl group in a similar position^27^, indicating that these molecules are capable of extensive binding site interactions in the ‘vaulted’ space of the interfacial GABA binding site revealed by our homology models of the GABA_A_ receptor. Previous studies of the GABA partial agonist, 4-PIOL, have also showed the cavernous nature of the GABA binding site, by accommodating large aromatic analogues with increased apparent binding affinity^52^. Possibly the hydrophobic nature of 4-PIOL^53^ and our gabazine analogues, may facilitate hydrophobic interactions (for example, π–π stacking) in the GABA binding site, which is lined with a number of aromatic side-chains.

The extended benzophenone analogue, GZ-B1, proved the most effective at irreversibly blocking α1β2γ2 GABA_A_ receptors following UV photoactivation, with near-saturating concentrations blocking ~50% of GABA_A_ receptors in an irreversible manner after only one cycle of UV. Although submaximal, this is more than sufficient for functional and trafficking studies of GABA_A_ receptors^15^. A similar level of inhibition was also reported for the photoactive glutamate receptor inhibitor, ANQX, on AMPA receptors^54^. However, for experiments that demand complete inhibition of GABA currents, several cycles of UV exposure can achieve this; although synaptic GABA currents can be virtually abolished by very few cycles of UV activation of GZ-B1. The reason why the block becomes more effective with successive UV exposure, most likely relates to the photochemical properties of the benzophenone group, which, unlike the azide and diazirine groups, does not lose N_2_ upon photoexcitation and thus can readily revert back to its ground state. This feature is advantageous since it allows the benzophenone group to have repeated attempts at covalent binding during successive periods of photoactivation.

The GABA concentration–response curves with GZ-B1 after photoactivation revealed a non-competitive depression compared with the competitive inhibition noted with reversible binding of GZ-B1 in the absence of UV. This is the expected behaviour of an irreversible antagonist at the agonist binding site, whereupon the GABA EC_50_ remains largely unaffected.

Once Cys-loop receptor agonists, such as GABA, are accommodated at their binding site, loop C is proposed to close, capping the binding site^36^,^37^,^55^, whereas no movement of loop C is observed with larger ligands of comparable size to gabazine and GZ-B1 (ref. 36)^36^. For the GZ-B1 molecule, computational docking analysis revealed that the benzophenone group extends along the β–α subunit interface to a region outside the recognized GABA binding site. Interestingly, aligning the primary sequences of α and β subunits along this part of the interface identified a lack of homogeneity for the α-subunits (Supplementary Fig. 5), which could underlie the slightly different potencies of GZ-B1 at some GABA_A_ receptors. However, the activity of GZ-B1 at both synaptic- and extrasynaptic-type GABA_A_ receptors suggests it can be considered as a broad spectrum photoactive antagonist.

The accuracy of our computational docking models for GABA, gabazine and GZ-B1 was affirmed by identifying α1-R119, β2-E155 and β2-R207 as key interacting residues in the GABA site, which have been previously reported^39^,^40^,^42^,^43^,^44^,^45^,^46^,^47^. This enabled the positioning of GZ-B1 within the binding site, and by further docking studies, the identification of new residues, α1-R84, β2-D162, β2-D163, and potentially α1-R119, as interactors with the benzophenone group.

While mutating these residues did not affect GABA binding, they were important for the reversible binding of GZ-B1, since a combined mutation, α1-R84Q and β2-R207Q caused a >1,000-fold loss of potency. We identified α1-R84 as the most important binding partner for the UV-activated GZ-B1 molecule, over β2-D162, β2-D163 and α1-R119. This suggests that GZ-B1 is optimally irreversibly bound in just one conformation at the binding site, with suboptimal binding conformations occasionally adopted. However, we should emphasize that docking solutions represent energy-minimized snapshots of the most prevalent three-dimensional (3D) orientations of the bound ligand. Nevertheless, the bound ligand, as well as the amino-acid side-chains at the binding site, will be constantly undergoing Brownian motion-like movement during covalent binding of GZ-B1. Thus, while the photoactivated benzophenone may, most commonly, associate with α1-R84, it could, at different times, associate with α1-R119, β2-D162 or β2-D163. These residues may play key roles in the energy-minimized positioning of GZ-B1 at the binding site, that is, by controlling the efficiency of the covalent attachment.

Applying GZ-B1 to cerebellar granule cells indicated that synaptic GABA_A_ receptors are very susceptible to inhibition and that this inhibition was irreversible over the time course of our recordings (usually >40 min). The level of inhibition was higher than that for whole-cell GABA currents. However, this does not involve changes to the affinity of the antagonist for the GABA_A_ receptors. By simulating synaptic and whole-cell GABA currents, the brief GABA concentration transient (~1 ms) and synaptic receptor occupancy expected at inhibitory synapses resulted in a higher level of block compared with that for longer whole-cell applications (~4 s) and correspondingly longer duration receptor occupancies.

In conclusion, by generating a new photoactivated gabazine analogue, GZ-B1, we can use UV photoactivation to irreversibly inactivate native GABA_A_ receptors both within and outside inhibitory synapses in addition to studying their trafficking without the need to having to use expression-tagged recombinant receptors or antibody-based labelling procedures. By determining where the photoactivated molecule is likely to bind, we have also mapped residues in a new region of the interface between β and α subunits just above the GABA binding site.

## Methods

### cDNA constructs

Murine α1 and β2 subunits and all point mutants were cloned into the plasmid pRK5, and verified by full-insert sequencing.

### Cell culture and expression of recombinant GABA_A_ receptors

HEK cells (ATCC, UK) were maintained in Dulbecco's Modified Eagle's Medium supplemented with 10% v/v fetal calf serum, 200 mM L-glutamine and 100 U ml^−1^ of penicillin/Streptomycin at 37 °C (95% air/5% CO_2_). Cells were plated onto poly-L-lysine coverslips and transfected with cDNAs encoding enhanced green fluorescent protein and murine α1-6, β1-3, γ2S, δ, ε and/or θ GABA_A_ receptor subunits using a calcium–phosphate method. Cells were used for electrophysiology experiments after 16–48 h (ref. 34).

Dissociated neuronal cultures were prepared from (E18-P4) Sprague–Dawley rats in accordance with UK Home Office regulations. Tissue blocks were incubated in trypsin for 10 min (0.1% w/v), washed in HBSS and then triturated in DNase (0.05% w/v in 12 mM MgSO_4_). Cells were plated on poly-L-ornithine-coated glass coverslips and cerebellar neurons were maintained in Basal Medium Eagle supplemented with 0.5% (w/v) glucose, 5 mg l^−1^ insulin, 5 mg l^−1^ transferrin, 5 mg l^−1^ selenium, 20 U ml^−1^ penicillin G and 20 μg ml^−1^ streptomycin, 0.2 mM glutamine, 1.2 mM NaCl and 5% (v/v) fetal calf serum. Hippocampal neurons were maintained in Neurobasal A supplemented with 1% v/v B-27, 50 U ml^−1^ penicillin-G and 50 μg ml^−1^ streptomycin, 0.5% v/v Glutamax, and 35mM glucose before transfection using a calcium phosphate-based method.

### Chemistry of gabazine analogues

To synthesize the photoreactive analogues, we developed a highly concise general strategy (Supplementary Fig. 1a). Suzuki–Miyaura coupling of 4-hydroxybenzeneboronic acid (referred to as ‘**1’** in Supplementary Fig. 1a) with 3-amino-6-chloropyridazine afforded a biaryl building block (**2**)^27^, which could then be reacted with the appropriate benzyl bromide to attach the photoactivatable groups. Finally, *N*-alkylation and mild deprotection of the allyl group afforded the products (**3**; either GZ-A1,-B1 or -D1) in just 4 steps and with good overall yields. The only exception to this strategy involved the synthesis of the truncated analogue GZ-B2, in which the boronic acid of the benzophenone was used directly, resulting in just a 3-step synthesis (Supplementary Fig. 1b; Supplementary Table 3, Supplementary Information—Chemistry).

### Electrophysiology and UV photoactivation

Whole-cell currents and sIPSCs were recorded from cells voltage clamped at −60 mV using an Axopatch 200B amplifier (Molecular Devices). Currents were filtered at 5 kHz (−3dB, 8 pole Bessel, 48 dB per octave) and digitized at 50 kHz via a Digidata 1320A (Molecular Devices) and recorded direct to a hard drive. Patch pipettes with a resistance of either 3–4 MΩ (HEK cells) or 8–9 MΩ (cerebellar granule cells) were filled with an intracellular solution containing (mM): 140 CsCl, 2 NaCl, 2 MgCl_2_, 5 EGTA, 10 HEPES, 0.5 CaCl_2_, 2 Na-ATP, 0.5 Na-GTP and 2 QX-314; pH 7 (adjusted with 1 M caesium hydroxide). Cells were continuously perfused with Krebs solution containing (mM): 140 NaCl, 4.7 KCl, 1.2 MgCl_2_, 2.52 CaCl_2_, 11 Glucose and 5 HEPES; pH 7.4 (adjusted with 1 M NaOH). In cerebellar granule cell experiments, the Krebs solution contained CNQX (10 μM) and AP5 (20 μM) to inhibit excitatory synaptic currents dependent on glutamate receptors. Drugs were applied to cells using a U-tube application system^56^.

Photoactivation was performed using a Rapp OptoElectronic JML-C2, with a band-pass filter of 240–400 nm and an optic fibre located in the bath 1–2 mm from the recorded cell. A single cycle of an optimized photoactivation protocol consisted of 10 flashes (2-s interval), capacitance 2 setting (C2) at 150 V. After UV exposure in the presence of the antagonist, the cell was left to rest for 3–5 min while washing with recording solution, to ensure that only covalently bound antagonist would remain in the binding site.

### Analysis of whole-cell current data

GABA concentration–response relationships were analysed by normalizing GABA currents to the response induced by a maximal, saturating GABA concentration (*I*_max_) and subsequently fitting with the Hill equation:


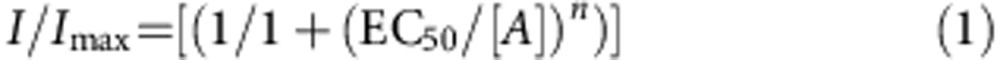


where EC_50_ represents the concentration of the agonist ([*A*]) inducing 50% of the maximal current evoked by a saturating concentration of the agonist and *n* represents the Hill coefficient.

Antagonists were evaluated for their potency by constructing inhibition–concentration relationship curves and fitting the data using:


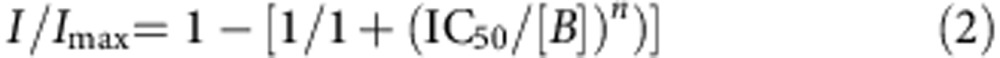


where the IC_50_ is the antagonist concentration ([*B*]) causing half-maximal inhibition of the GABA (EC_50_)-induced response. When complete inhibition was not attained, the above equation was modified to:


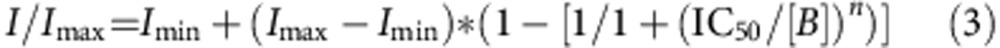


where *I*_min_ represents the residual GABA current remaining with a saturating concentration of antagonist, and *I*_max_ represents the control peak GABA-activated current.

The IC_50_ values obtained from individual experiments were converted to pIC_50_ values (=−Log (IC_50_). Mean pIC_50_ values±s.e.m. of at least four experiments were subject to statistical analyses (ANOVA and Student’s *t*-test). Potency histograms have two *y* axes for mean pIC_50_ values±s.e.m., and the IC_50_ transform (note: error bars refer only to the pIC_50_).

The competitive antagonism caused by gabazine and its analogues was analysed according to the Schild method^31^. Full GABA concentration–response curves were obtained in control Krebs in each HEK cell and then one or more curves were established in up to four concentrations of gabazine or one of its analogues. The curves were tested for parallelity and the dose ratios for GABA were calculated from the respective GABA EC_50_s. The mean dose ratios for each antagonist concentration (*B*) allowed the dissociation constant (*k*_B_) to be determined using the transformed Schild equation:


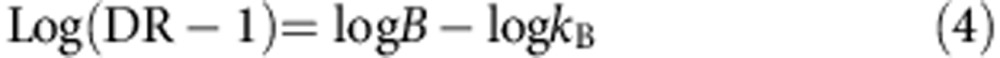


The slope of the Schild plot (log (DR−1) versus log [B]) was tested to ensure its slope did not deviate significantly from unity. The slopes were then constrained to 1 and the intercept on the abscissa (‘dose ratio—1’) was used to ascertain the pA_2_ (= −log *k*_B_).

The level of spontaneous activity observed with mutant GABA_A_ receptors containing the β2^E155Q^ mutation was determined as, the maximal inhibition of channel activity observed in the presence of 1 mM picrotoxin (*I*_PTX−max_), divided by the total range of channel activity (*I*_PTX−max_+*I*_GABA−max_) (ref. 57).

### Homology modelling and computational docking

Murine α1, β2, γ2 subunits were aligned to the subunit sequence of AChBP and GluCl using the T-COFFEE server^58^ with manual adjustment. Based on the alignment, two 3D homology models of the α1β2γ2 GABA_A_ receptor were built with MODELLER^59^ using the crystal structures of AChBP (PDB ID: 2BYN) at 2.02 Å resolution and of GluCl (PDB ID: 3RHW) at 3.26 Å resolution. The GABA_A_ receptor α1 subunit exhibits 22% and 31% sequence identity with those of AChBP and GluCl, respectively. In comparison with AChBP and GluCl, the GABA_A_ receptor β2 subunit shares 22% and 36% sequence identity.

Initially, our docking studies were performed on the GABA_A_ receptor homology model derived from AChBP. First, GABA, gabazine and GZ-B1 were docked into the GABA binding site of the homology model. The binding site cavity was defined such that all the receptor residues defined within a sphere of 10 Å radius from the α-carbon of Y157 (β2) were included. Hermes version 1.4.1 interface and GOLD version 5.0.1 (ref. 35)^35^ were used to initiate docking. The genetic algorithm settings in GOLD were automatically optimized with maximum search efficiency. During the first stage, all the ligands were docked into the binding site and were kept fully flexible during docking. Ten residues within the binding cavity were selected and their side-chains were allowed full flexibility during docking: F64 (α1), R66 (α1), R119 (α1), Y97 (β2), F98 (β2), E155 (β2), Y157 (β2), Y159 (β2), F200 (β2), Y205 (β2) and R207 (β2). For each of the ligands, 50 diverse docking solutions were generated using the GoldScore scoring function with default parameters. From our homology models, we identified a new cavity at the β–α subunit interface (located higher up than the GABA binding site), which could feasibly accommodate large ligands. To further explore the potential binding residues found in the new cavity, we performed a second stage of docking only for the GZ-B1 case, using GOLD with the ‘scaffold-match constraint’ (starting from the selected binding mode obtained from the first stage of docking without any constraints). The scaffold-match constraint was used to maintain a fragment at an exact specified position in the binding site with the geometry of this fragment remaining unaltered during docking. All the atoms in GZ-B1 molecule, except the benzophenone group, were retained as a scaffold.

To investigate the new binding cavity, we performed further docking using a separate ‘region constraint’. This was used to bias the docking solutions towards a particular region of the binding site, constraining specific ligand atoms in this region. For this constraint, the centroid of the residues defining the orifice of the new cavity (R84 (α1), L85 (α1), N87 (α1), F31 (β2), D162 (β2) and D163 (β2)) was calculated with Chimera^60^ and the binding site region was defined within a sphere of 5 Å radius around this centroid. All the benzophenone atoms of GZ-B1 were constrained to occupy the new binding site region. The receptor residues in and around the new cavity (R84 (α1), L85 (α1), N87 (α1), R119 (α1), F31 (β2), Y159 (β2), T160 (β2), D162 (β2), D163 (β2) and Y205 (β2)) were allowed full flexibility during the docking runs.

All the docking studies on GZ-B1 described above (two-stage docking and region-constraint docking) were also applied to the GABA_A_ receptor homology model derived from GluCl. For the two-stage docking, we included an ‘H-bond constraint’ in addition to a scaffold-match constraint. The new constraint was added to promote H-bond interaction between the acceptor oxygen atom of the benzophenone in GZ-B1 and the donor nitrogen atoms of side-chain of R84 found in the newly identified cavity.

For analysing the results, all the H-bond interactions were identified using GOLD. We also analysed cation–π interactions, which are considered to be important for drug–receptor binding and are energetically comparable to H-bond interactions^61^. If the distance between the cation and the centroid of the π system is within 6 Å, and the angle between the line joining the cation, and that the centroid and the normal to the aromatic plane at the centroid is between 0 and 90°, we accepted this as a cation–π interaction^62^. The r.m.s.d. was used as a measure to compare different binding modes. For r.m.s.d. calculation, we only used the scaffold atoms of gabazine and GZ-B1 (those forming the rings and connecting them). Two binding modes with r.m.s.d. less than or equal to 2.5 Å were considered to be similar.

### Tracking GABA receptor mobility

The mobilities of GABA_A_ receptors in cultured hippocampal neurons were tracked using QDs photo-linked to GABA_A_ receptors via GZ-B1–biotin (see legend to Supplementary Fig. 4). Cells were treated with 0.5 mM GZ-B1–biotin (previously incubated for 3 min with 25 pM QD_655_–streptavidin; Life Technologies) and either not exposed (control) or UV exposed (40 s) followed by washing of cells in Krebs solution.

Mobilities were also studied using GABA_A_ α1 subunits tagged with QDs via a primary antibody against α1 (gift from Jean-Marc Fritschy, Zurich; incubation in 1 μg ml^−1^ for 2 min) and a secondary antibody containing biotin (Millipore; incubation in 5 μg ml^−1^ for 2 min) and QD_655_–streptavidin (25 pM; 1 min incubation). Trajectories were analysed using the ImageJ plug-in, SpotTracker 2D/3D and MatLab.

## Author contributions

J.R.B., F.I., R.H., M.M. and T.G.S. designed the gabazine analogues and F.I. and R.H. undertook their synthesis; M.M. made receptor mutations and performed all electrophysiology and photolysis experiments on recombinant receptors. A.P.P. and M.T. designed, performed and analysed the modelling work. S.H., M.M. and R.H. performed the quantum dot experiments and S.H. analysed the data. M.M. and T.G.S. designed the project, the experiments and analysed data, interpreting the results and wrote the paper. All authors contributed to the writing of the paper.

## Additional information

**How to cite this article:** Mortensen, M. *et al.* Photo-antagonism of the GABA_A_ receptor. *Nat. Commun.* 5:4454 doi: 10.1038/ncomms5454 (2014).

## Supplementary Material

Supplementary Figures, Tables, Methods and References Supplementary Figures 1-5, Supplementary Tables 1-3, Supplementary Methods and Supplementary References

Supplementary Movie 1 Internalisation of GZ-B1-biotin-streptavidin-QD-labelled GABA_A_ receptors in hippocampal neurons
