# Supplementary material for: Photo-antagonism of the GABAA receptor
Source: Nat Commun. 2014 Jul 29;5:4454. doi: 10.1038/ncomms5454 (PMC4124873; doi:10.1038/ncomms5454)
Supplement: Supplementary Figures, Tables, Methods and References — Supplementary Figures 1-5, Supplementary Tables 1-3, Supplementary Methods and Supplementary References [file ncomms5454-s1.pdf]

Supplementary Figure 1

a

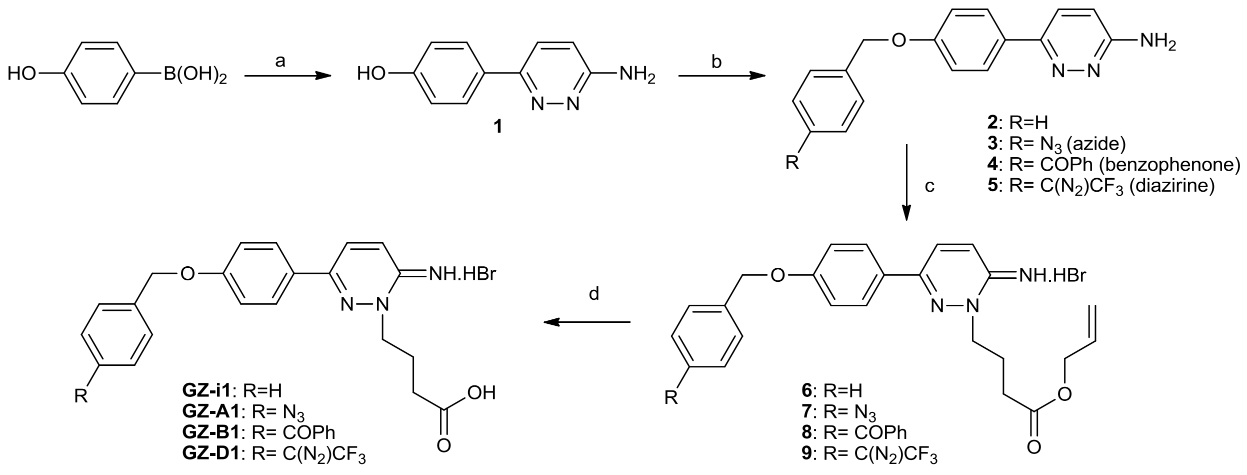

Reagents and conditions: a) 3-amino-6-chloropyridazine, $Pd(PPh_3)_2Cl_2$, K_2CO_3, CH_3CN/H_2O (3:2), microwave (10 min, 120 ^oC), 72 %; b) R-benzyl bromide, KO^tBu, DMF (3 h, 0 ^oC), 31-62 %; c) allyl-4-bromobutyrate, DMF (5-17 h, 80 ^oC), 37-85 %; d) $Pd(PPh_3)_4$, scavenger ligand, THF/CH_3OH (4:1), 56-94 %. Full synthetic procedures are outlined in Supplementary Information.

b

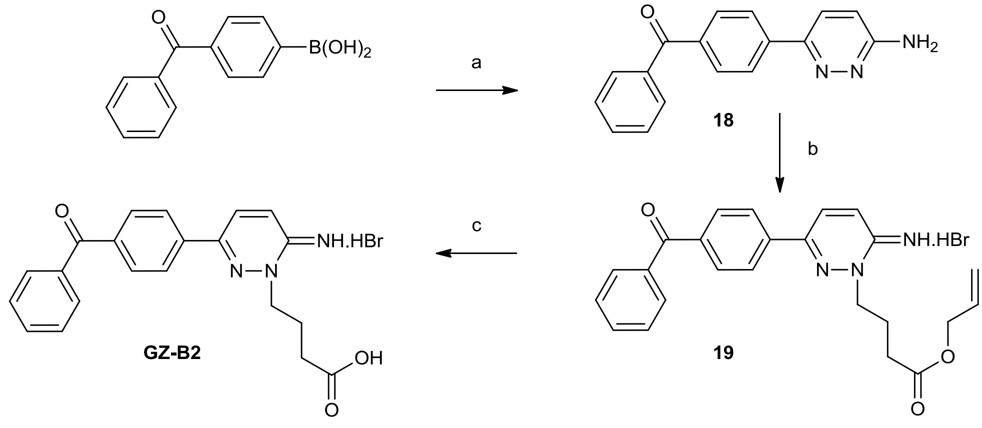

Reagents and conditions: a) 3-amino-6-chloropyridazine, $Pd(PPh_3)_2Cl_2$, K_2CO_3, CH_3CN/H_2O (3:2), microwave (10 min, 120 ^oC), 61 %; b) allyl-4-bromobutyrate, DMF (18 h, 80 ^oC), 76 %; c) $Pd(PPh_3)_4$, morpholine, THF/CH_3OH (4:1) (30 min, rt), 92 %. Full synthetic procedures are outlined in Supplementary Information.

c

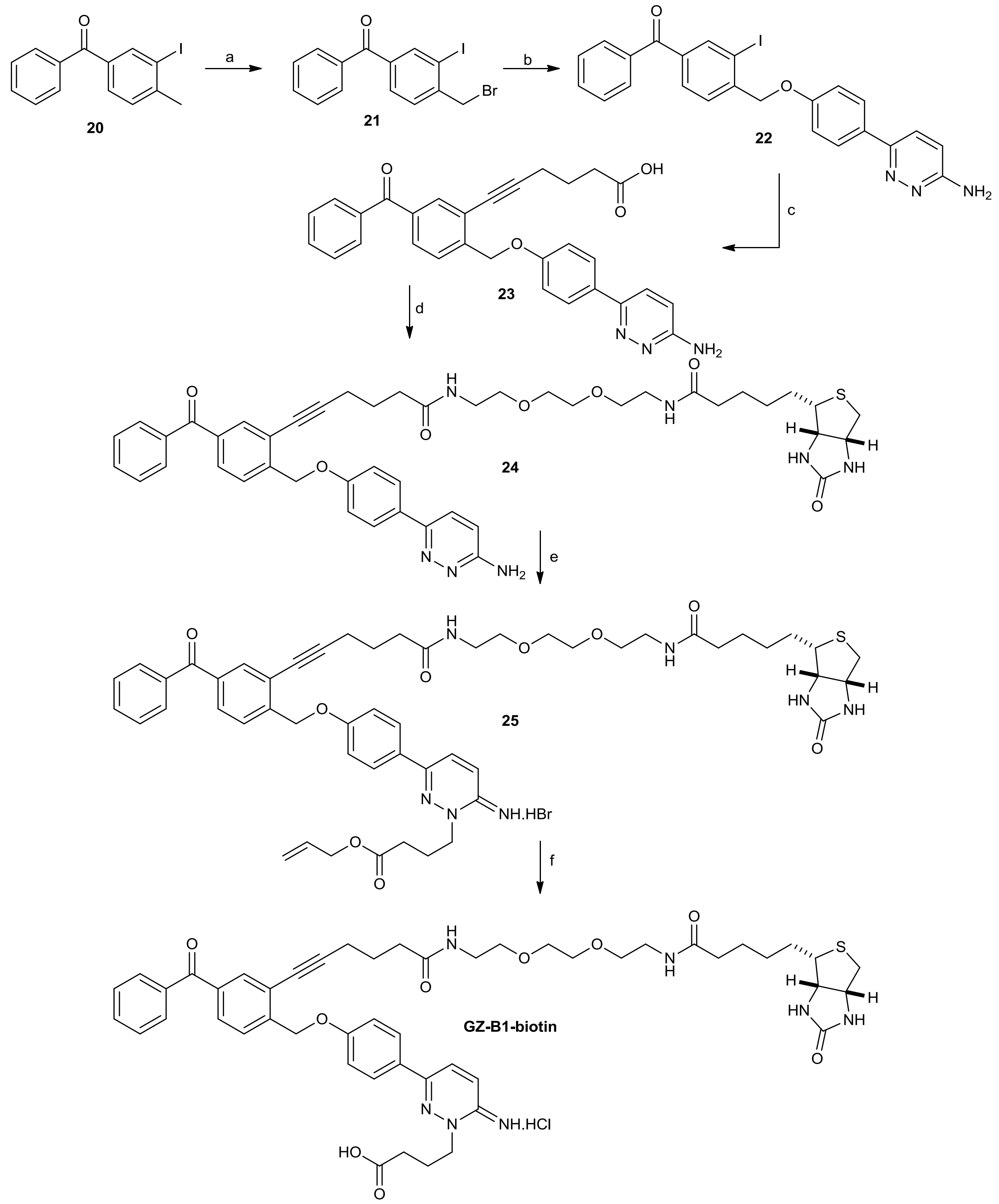

Reagents and conditions: a) NBS, AIBN, benzene (16 h, 80°C), 56%; b) NaH, **1**, DMF (4 h, 0 - 20 °C), 82%; c) 5-Hexynoic acid, $Pd(PPh_3)_3$, CuI, Et_3N, THF (16 h, 65 °C), 49%; d) DCC, *amine* **28** , Et_3N, DMF (16 h, rt), 52%; e) Allyl 4-bromobutyrate, DMF (16 h, 120 °C), 77%; f) NaOH, THF, H_2O (3 h, 50°C); HCl, H_2O (1 h, 0 °C) 65%. Full synthetic procedures are outlined in Supplementary Information.

Supplementary Figure 1 Organic synthesis

(**a**) Synthesis of the gabazine analogues: GZ-A1, GZ-B1 and GZ-D1. (**b**) Synthesis of the truncated analogue GZ-B2. (**c**) Synthesis of biotinylated gabazine analogue GZ-B1-biotin. (See supplemental information for full experimental procedures).

Supplementary Figure 2

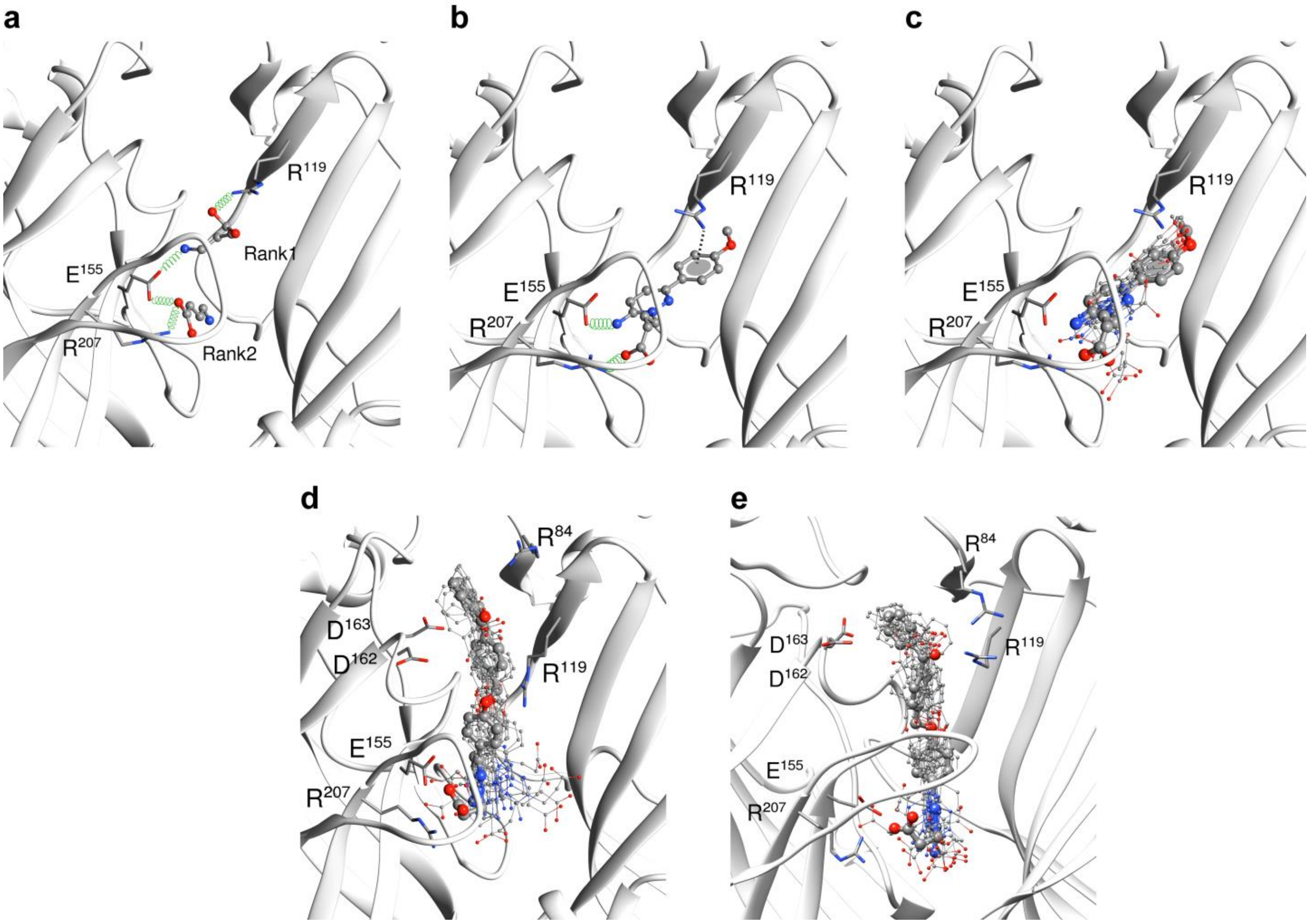

Supplementary Figure 2 Docking GABA, gabazine and GZ-B1 at the binding site

GABA binding site model showing computational docking of GABA and gabazine to identify critical binding residues. (**a**) The top two predicted binding modes for GABA (Ranks 1 and 2) are shown at the GABA binding site. Rank 1 forms H-bonds with R119 (α1) and E155 (β2), whereas rank 2 represents a potential alternate binding mode involving H-bonds with R207 (β2) and E155 (β2). (**b**) Predicted binding mode for gabazine. The carboxyl group of gabazine is predicted to H-bond with R207 (β2) and E155 (β2), and the aromatic ring is predicted to form a cation-π interaction with R119 (α1). (**c**) Cluster of gabazine binding modes. The predicted binding mode (**b**) is shown as a large ball and stick representation along with similar binding modes (11 out of 50 shown as small ball and stick representations) based on RMSD measures (See Methods). (**d**) Cluster of GZ-B1 binding modes based on AChBP. The predicted binding mode is shown as a large ball and stick representation along with similar binding modes (13 out of 50 shown as small ball and stick representations). (**e**) Cluster of GZ-B1 binding modes based on GluCl. The predicted binding mode is shown in large ball and stick format along with similar binding modes (15 out of 50 shown in small ball and stick format). H-bonds are depicted as coloured dashed lines, with cation-π interactions as dashed lines in black. The subunits are shown in ribbon format.

Supplementary Figure 3

a

						LoopB	
β1	26	RLRPDFG	32		158	GYTTDD	163
β2	26	RLRPDFG	32		158	GYTTDD	163
β3	26	RLRPDFG	32		158	GYTTDD	163

b

						LoopE	
α1	82	VLRLNNLM	89		117	LLRITE	122
α2	82	ILRLNNLM	89		117	LLRIQD	122
α3	107	ILPLNNLL	114		142	LLRLVD	147
α4	81	ILRLNNMM	88		116	LFRIMR	121
α5	92	RLPLNNLL	99		127	LLRLED	132
α6	81	ILSLNNLM	88		116	LFRLMQ	121

Supplementary Figure 3 Binding site residues aligning with the benzophenone group

(**a**) Primary sequence alignments of $GABA_A$ receptor β1-3 subunits from two stretches of residues predicted to oppose the benzophenone group of GZ-B1. The latter contains loop B. (**b**) Similar alignments of residues in α1-6 subunits opposing the benzophenone group. The latter contains loop E. Boxed areas indicate conserved residues. In α-subunits residues have been colour codes to highlight amino acid differences.

Supplementary Figure 4

a

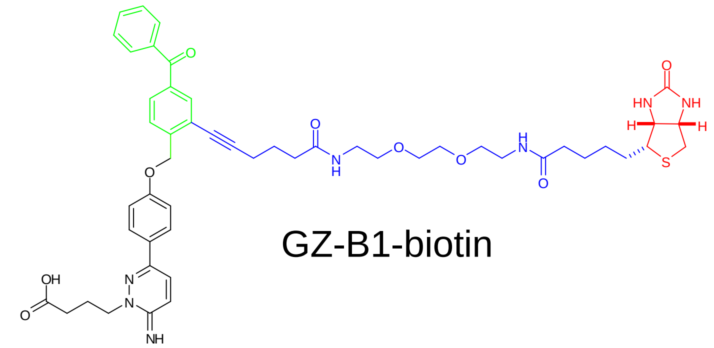

b

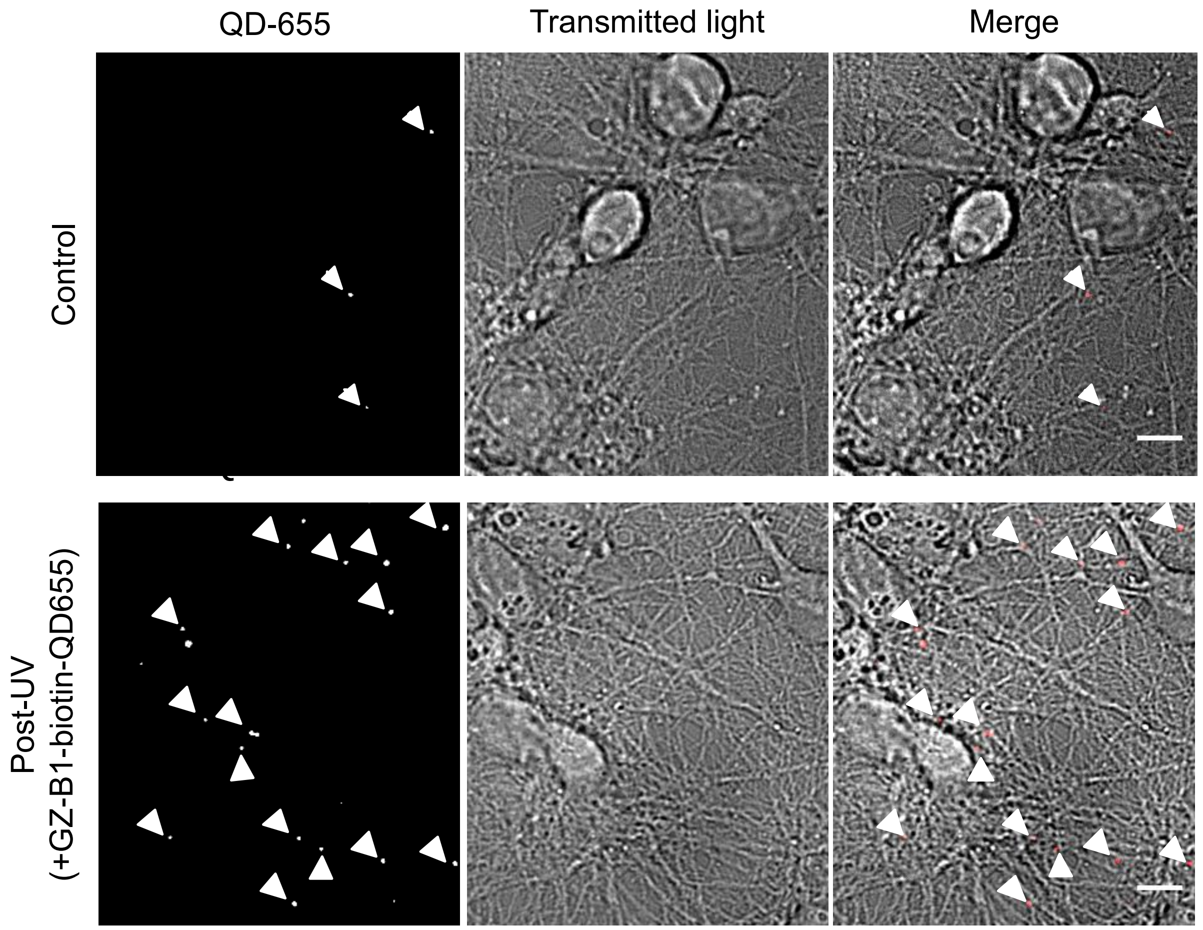

c

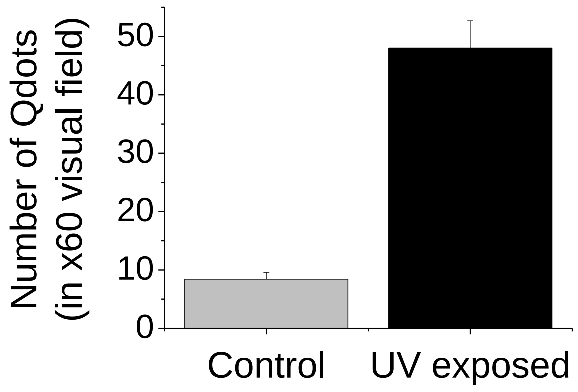

d

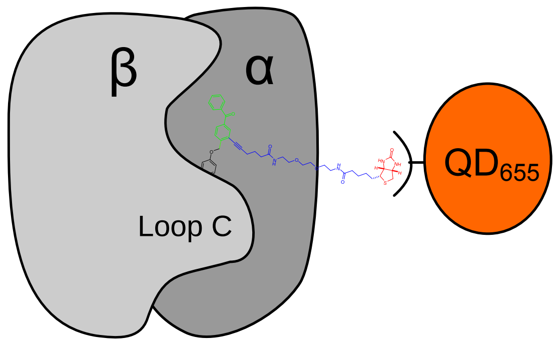

Supplementary Figure 4 Quantum dot binding to hippocampal neurons via GZ-B1-biotin

(**a**) Structure of GZ-B1-biotin. Colour coded groups of the molecule are: gabazine (black), benzophenone (green), alkene and polyethylene glycol (PEG) linker (blue), biotin (red). (**b**) Cultured hippocampal neurons shown after treatment with 0.5 mM GZ-B1-biotin (previously incubated for 3 min with 25 pM QD_{655}-streptavidin; Life Technologies) not-exposed (control) or UV exposed (40 s) followed by washing of cells in Krebs solution. DIC and quantum dot fluorescent (655 nm) images are shown. Note the significantly greater number of bound QDs observed in the UV treated dishes. Scale bar: 20 μm. (**c**) Histogram showing significantly higher specific QD labelling in UV treated dishes (n = 4). (**d**) Schematic representation of the proposed orientation of a bound GZ-B1-biotin molecule at the GABA binding site. The gabazine group (out of view) nestles behind Loop C of the β subunit with the benzophenone group largely above. This allows the PEG and biotin groups to orientate away from the $GABA_A$ receptor facilitating a strong bond with a streptavidin-coated quantum dot.

Supplementary Figure 5

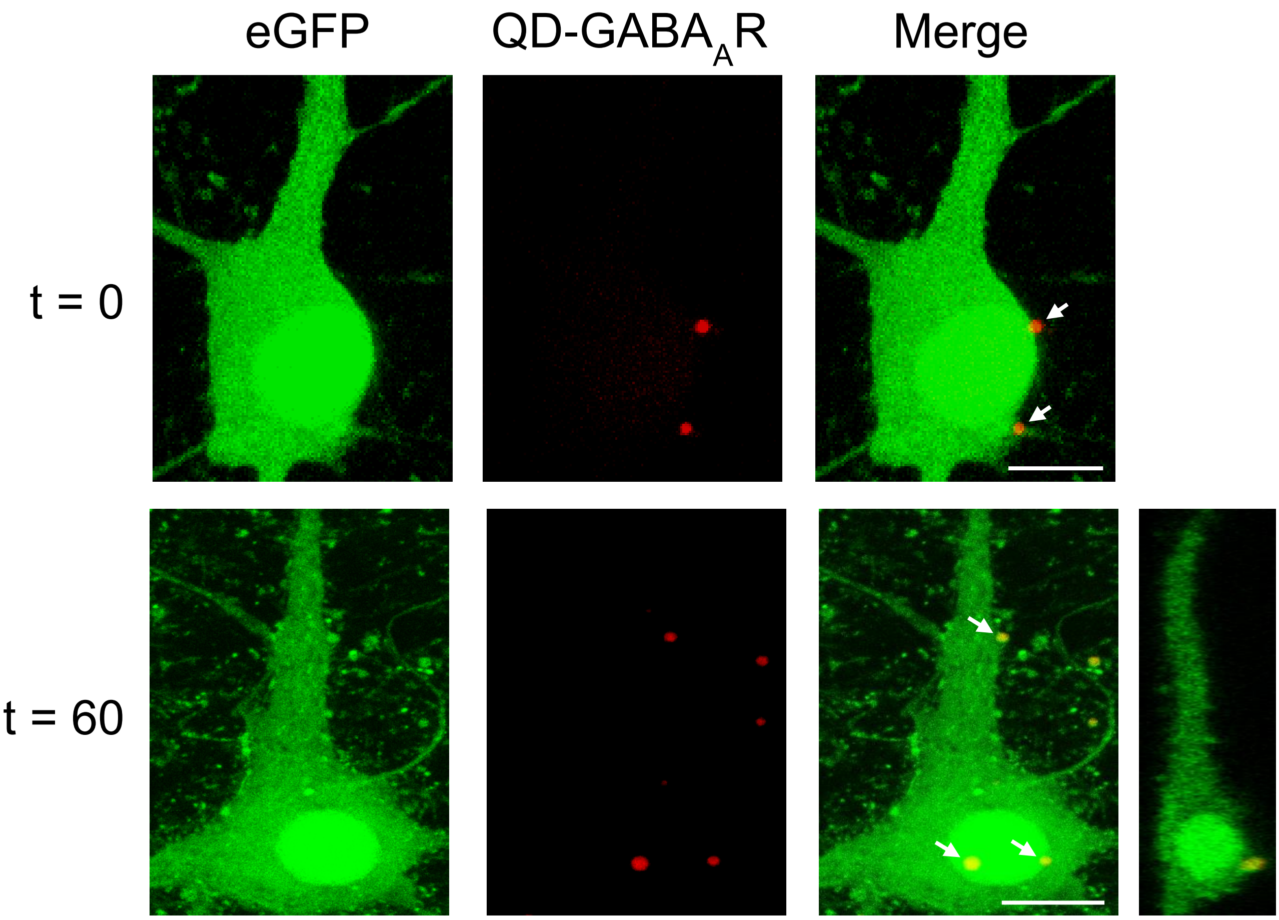

Supplementary Figure 5 Internalisation of QD-labelled GABA$_A$ receptors in hippocampal neurons

At 7 DIV, hippocampal neurons were transiently transfected with eGFP cDNA using a calcium phosphate method to enable visualisation of the cell bodies, dendritic and axonic processes. Approximately 1 week later, neurons were incubated with 0.5 mM GZ-B1-biotin (pre-reacted with 25 pM QD_{655} for 3 min), then UV exposed for 40 s, and washed with Krebs solution. Fixation was carried out at t = 0 min or after incubation for 1 hr at 37°C (t = 60 min). The images are maximum intensity projections of z-stacks (left and right panels) from hippocampal neurons at 12-14 DIV expressing eGFP labelled with GZ-B1-biotin-streptavidin-QD_{655}. The QDs are shown as red dots in the middle panel. A Y-Z axis projection (lower panel, far right) has been included at t = 60 to show the location of the internalised QDs in the cytosol between the nucleus and the plasma membrane. Images were acquired using a SP8vis confocal microscope with a resonant scanner, x40 oil objective, 405 nm excitation for QD655 and 488 nm for eGFP. Images were processed using Fiji (ImageJ v.1.48). Scale bar = 10 µm. Arrows indicate surface (t = 0) or internalised QDs (t = 60). For a 3D projection see **Supplementary Movie 1**.

Supplementary Table 1

Functional effects of binding site mutations for GABA, gabazine and GZ-B1

Isoform	Spont. activity	GABA pEC50 (EC50)	Gabazine pIC50 (IC50) Max inhibition	GZ-B1 pIC50 (IC50) Max inhibition	GABA max currents (pA pF^{-1})
α1β2γ2 (wt)	n.d.	5.180 ± 0.0593 (34)<br>(6.6 *µM*)	6.457 ± 0.0308 (8)<br>(0.3491 *µM*)<br>100 %	6.809 ± 0.0852 (6)<br>(0.1553 *µM*)<br>100 %	221 ± 25 (13)
α1^{R84Q}β2γ2	n.d.	4.776 ± 0.0817 (6)<br>(17 *µM*)	n.t.	5.374 ± 0.0756 (5)<br>(4.22 *µM*)<br>100 %	242 ± 32 (6)
α1^{R119Q}β2γ2	n.d.	3.809 ± 0.1484 (8)<br>(155 *µM*)	6.727 ± 0.2809 (7)<br>(0.1876 *µM*)<br>100%	7.146 ± 0.2142 (5)<br>(0.0715 *µM*)<br>100 %	207 ± 55 (8)
α1β2^{R207Q}γ2	n.d.	3.344 ± 0.1241 (6)<br>(452 *µM*)	5.768 ± 0.0885 (5)<br>(1.7077 *µM*)<br>100 %	6.312 ± 0.0303 (5)<br>(0.4871 *µM*)<br>83 ± 3 %	249 ± 48 (6)
α1β2^{E155Q}γ2	72 ± 3 %	2.580 ± 0.3561 (6)<br>(2,628 *µM*)	<4 (4)^{i}<br>(>100 *µM*)<br>n.d.	<4 (4)^{i}<br>(>100 *µM*)<br>n.d.	25 ± 6 (6)
α1β2$^{D162+163N}$γ2	n.d.	4.752 ± 0.0589 (5)<br>(18 *µM*)	n.t.	4.869 ± 0.0933 (5)<br>(13.5 *µM*)<br>n.d.	175 ± 27 (5)
α1β2$^{E155Q+R207Q}$γ2	7 ± 1 %	3.326 ± 0.0674 (6)<br>(473 *µM*)	5.860 ± 0.3535 (5)<br>(1.3817 *µM*)<br>100 %	6.328 ± 0.0526 (5)<br>(0.4700 *µM*)<br>100 %	152 ± 32 (6)
α1^{R119Q}β2^{E155Q}γ2	87 ± 3 %	3.113 ± 0.263 (4)<br>(772 *µM*)	<4 (4)^{i}<br>(>100 *µM*)<br>n.d.	<4 (4)^{i}<br>(>100 *µM*)<br>n.d.	32 ± 16 (4)
α1^{R84Q}β2^{R207Q}γ2	n.d.	3.020 ± 0.2515 (5)<br>(955 *µM*)	n.t.	3.740 ± 0.1224 (6)<br>(182 *µM*)<br>n.d.	124 ± 39 (5)
α1^{R119Q}β2^{R207Q}γ2	n.d.	1.799 ± 0.2616 (6)<br>(15,885 *µM*)	5.426 ± 0.2565 (5)<br>(3.7535 *µM*)<br>100 %	6.379 ± 0.0422 (5)<br>(0.4182 *µM*)<br>75 ± 2 %	120 ± 27 (6)
α1^{R119Q}β2$^{E155Q+R207Q}$γ2	10 ± 3 %	1.746 ± 0.1982 (6)<br>(17,968 *µM*)	5.645 ± 0.0579 (5)<br>(2.2623 *µM*)<br>94 ± 1 %	6.371 ± 0.1654 (5)<br>(0.3381 *µM*)<br>72 ± 4 %	81 ± 23 (6)
α1$^{R84+119Q}$β2$^{D162+163N}$γ2	n.d.	2.032 ± 0.022 (5)<br>(9,282 *µM*)	n.t.	3.589 ± 0.1027 (5)<br>(258 *µM*)<br>n.d.	83 ± 25 (5)

Supplementary Table 1: Potencies of GABA and antagonists including extent of spontaneous channel opening for wild-type and mutant $GABA_A$ receptors. n.d. not detectable; n.t. Not tested; i: inhibition of spontaneous activity, since agonist induced responses (EC_{50}) were too small. Maximum currents are shown as pA pF^{-1} (average cell capacitance: 13.4 ± 0.9 pF).

Supplementary Table 2

GABA potency before and after UV exposure in the presence of GZ-B1

Isoform	Conc. of GZ-B1 during UV (μM)	Pre-UV GABA pEC_{50} (EC_{50})	Post-UV GABA pEC_{50} (EC_{50})	P
α1β2γ2	10	5.259 ± 0.0913 (6) (5.51 *μM*)	5.542 ± 0.1026 (6) (2.87 *μM*)	0.06
$α1^{R119Q}β2γ2$	10	3.385 ± 0.1006 (6) (412 *μM*)	3.596 ± 0.04614 (6) (254 *μM*)	0.09
$α1β2^{D162N, D163N}γ2$	100	4.607 ± 0.0416 (4) (25 *μM*)	4.330 ± 0.1943 (4) (47 *μM*)	0.21
$α1^{R84Q}β2γ2$	100	4.514 ± 0.1377 (6) (31 *μM*)	4.541 ± 0.1228 (6) (29 *μM*)	0.88
$α1^{R84Q}β2^{R207Q}γ2$	100	3.020 ± 0.2515 (5) (955 *μM*)	2.932 ± 0.2530 (5) (1169 *μM*)	0.81
$α1^{R84Q, R119Q}β2^{D162N, D163N}γ2$	100	2.032 ± 0.022 (4) (9282 *μM*)	1.949 ± 0.0421 (4) (11240 *μM*)	0.11

Potencies of GZ-B1 measured for wild-type and mutant $GABA_A$ receptors before and after UV exposure

Supplementary Table 3

Partition coefficients for gabazine analogues

Compound	cLogP
GZ-A1	2.65
GZ-B1	3.49
GZ-D1	4.63
GZ-B2	2.22
GZ-B1-biotin	5.07

Supplementary Methods

All chemical reactions were carried out at atmospheric pressure with stirring unless otherwise stated. All reagents and solvents were purchased from suppliers and used without further purification unless otherwise stated. Thin-layer chromatography (TLC) was performed on aluminium-backed TLC plates pre-coated with Merck silica gel 60 F_{254}. Compounds were visualized with UV light and/or staining with $KMnO_4$ or vanillin. Flash column chromatography was performed using silica gel 60 (230-400 mesh). Solvents used for anhydrous reactions were dried and distilled immediately prior to use. For use in anhydrous reactions all glassware was flame-dried and cooled under an argon atmosphere immediately prior to use. NMR spectra were recorded on a Bruker 600 or 500 MHz spectrometer. Chemical shifts (δ) are listed in ppm downfield from TMS. Coupling constants are reported in Hz. High and low resolution mass spectrometry was performed using a VG70 SE operating in modes CI, EI, ES and FAB. Infrared spectra were obtained on a Perkin Elmer Spectrum 100 FTIR Spectrometer operating in ATR mode. UV/Vis absorbance spectra were recorded using a Cary WinUV spectrometer. Wavelength(s) corresponding to any absorbance maxima (λ_{max}) are given in nm, and molar extinction co-efficients (ε) are given in $M^{-1}cm^{-1}$.

UV - visual spectra

UV - visual spectum for photoaffinity labels **GZ-B1** and **GZ-B1-biotin**, indicating the presence of the n-π^* absorbance at λ = 360 nm.

GZ-B1

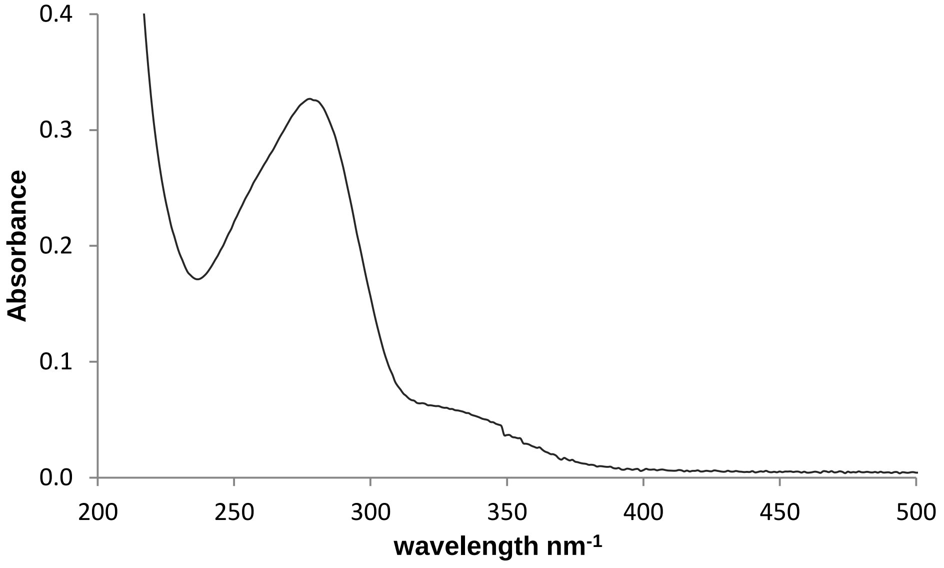

UV spectra of GZ-B1 (c= 2.0×10^{-5} M)

GZ-B1-biotin

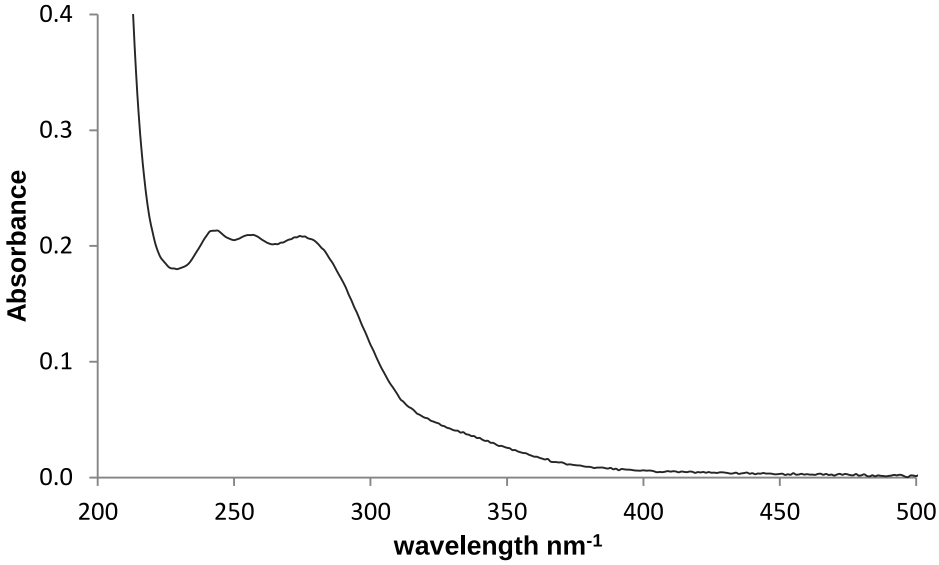

UV spectra of GZ-B1-biotin (c= $1.5x10^{-3}$ M).

Partition coefficients for the gabazine analogues were estimated using OSIRIS Property Explorer[1] to determine the partition coefficient between n-octanol and water (clogP = $\{log\left(\frac{C_{octanol}}{C_{water}}\right)\}$) (**Supplementary Table 3**). Melting points were measured with a Gallenkamp apparatus and are uncorrected. Room temperature (rt) is defined as between 19-22 ^{o}C. *In vacuo* is used to describe solvent removal by rotary evaporation between 20 ^{o}C and 60 ^{o}C, at approximately 10 mmHg unless otherwise stated. The term 'degassed' refers to the process of removing O_2 from a solution by bubbling argon through the solution prior to use. Microwave irradiation was carried out in a CEM 150W microwave reactor.

4-(6-Amino-pyridazin-3-yl)-phenol (**1**)

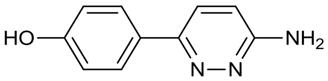

To a microwave vial containing 3-amino-6-chloropyridazine (102 mg, 0.740 mmol), 4-hydroxyphenylboronic acid (163 mg, 1.26 mmol), bis(triphenylphosphine)palladium(II) dichloride (27 mg, 0.040 mmol) and K_2CO_3 (202 mg, 1.46 mmol) were added CH_3CN (2.0 mL) and H_2O (1.3 mL). The resulting solution was degassed for 5 min and subjected to microwave irradiation for 10 min at 120 °C. The mixture was diluted with water (50 mL) and extracted with EtOAc (3 × 100 mL), washed with brine (100 mL), dried ($MgSO_4$) and concentrated *in vacuo*. The residue was purified by column chromatography (EtOAc:CH_3OH, 19:1 v/v) to give the *pyridazine* **1** (87 mg, 0.459 mmol, 62%) as an orange solid. m.p.: 250-252

°C; TLC (EtOAc): RF = 0.10; ^{1}H-NMR (CD_3OD:$CDCl_3$, 1:1 v/v, 600 MHz) δ 7.68 (d, *J* = 8.9 Hz, 2H), 7.64 (d, *J* = 9.3 Hz, 1H), 6.97 (d, *J* = 9.3 Hz, 1H), 6.88 (d, *J* = 8.9 Hz, 2H); ^{13}C-NMR (CD_3OD, 125 MHz) δ 159.3, 157.8, 150.0, 128.0, 126.7, 124.7, 115.5, 114.4; IR (film): 3414, 3121, 1646, 1617, 1447 cm^{-1}; HRMS ($m\ z^{-1}$): $[M]^+$ calculated for $C_9H_9N_3O$, 187.0740; found, 187.0732.

<u>Synthesis of GZ-i1</u>

6-(4-Benzyloxyphenyl)-pyridazin-3-yl amine (**2**)

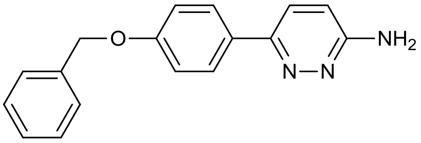

1 (250 mg, 1.34 mmol) and sodium hydride (54 mg, 1.34 mmol) in DMF (2 mL) was cooled to 0 °C. Benzyl bromide (239 mg, 1.40 mmol) was added dropwise and the reaction mixture was stirred at 0 °C for 2 h. The reaction mixture was extracted with Et_2O (100 mL) and washed with water (100 mL). The aqueous layer was further extracted with Et_2O (4 × 50 mL). The organic extracts were combined and dried ($MgSO_4$). The solvent was removed *in vacuo* and the residue subjected to column chromatography (EtOAc) to give the *pyridazine* **2** (151 mg, 0.544 mmol, 41%) as a white solid. m.p.: 190-192 °C; TLC (EtOAc:CH_3OH, 20:1 v/v): RF = 0.30; ^{1}H-NMR (CD_3OD, 500 MHz) δ 7.81(d, *J* = 6.8 Hz, 2H), 7.72 (d, *J* = 9.3 Hz, 1H), 7.44 (d, *J* = 7.3 Hz, 2H), 7.36 (dd, *J* = 7.3 and 7.2 Hz, 2H), 7.30 (t, *J* = 7.2 Hz, 1H), 7.08 (d, *J* = 6.8 Hz, 2H), 6.98 (d, *J* = 9.3 Hz, 1H), 5.13 (s, 2H); ^{13}C-NMR (CD_3OD, 125 MHz) δ 161.0, 138.5, 136.2, 130.7, 129.6, 129.0, 128.6, 128.5, 128.2, 127.9, 117.7, 116.3, 71.0; IR (film): 3431, 3282, 3113, 1647, 1609 cm^{-1}; HRMS ($m\ z^{-1}$): $[M]^+$ calculated for $C_{17}H_{15}N_3O$, 278.1281; found, 278.1293.

1-(3-Allyloxycarbonylpropyl)-6-amino-3-(4-benzyloxyphenyl)-pyridazinium bromide (**6**)

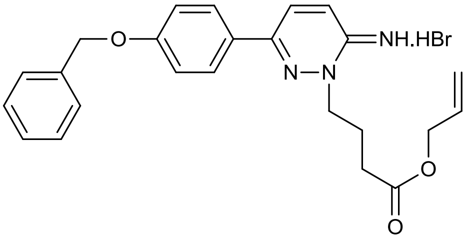

To a solution of **2** (50 mg, 0.180 mmol) in DMF (0.2 mL) was added allyl-4-bromobutyrate (56 mg, 0.271 mmol). The solution was heated to 80 °C for 16 h. The hot solution was poured into EtOAc (5 mL) to yield a solid, which was then isolated by filtration. The product was dried under high vacuum to give the *ester* **6** (51 mg, 0.105 mmol, 59%) as a grey solid. m.p.: 174-177 °C; TLC ($CHCl_3$:CH_3OH, 8:2 v/v): RF = 0.60; ^{1}H-NMR (CD_3OD, 500 MHz) δ 8.27 (d, *J* = 9.6 Hz, 1H), 7.93 (d, *J* = 5.6

Hz, 2H), 7.59 (d, *J* = 9.6 Hz, 1H), 7.44 (d, *J* = 7.4 Hz, 2H), 7.37 (dd, *J* = 7.4 and 7.1 Hz, 2H), 7.32 (t, *J* = 7.1 Hz, 1H), 7.13 (d, *J* = 5.6 Hz, 2H), 5.81 (m, 1H), 5.22 (dd, *J* = 15.7 and 1.5 Hz, 1H), 5.15 (s, 2H), 5.13 (dd, *J* = 8.0 and 1.5 Hz, 1H), 4.46-4.44 (m, 4H), 2.60 (t, *J* = 6.8 Hz, 2H), 2.19-2.14 (m, 2H); ^{13}C-NMR (CD_3OD, 125 MHz) δ 174.0, 162.7, 153.9, 151.9, 138.2, 133.4, 132.6, 129.6, 129.3, 129.1, 128.6, 126.7, 126.6, 118.6, 116.7, 71.1, 66.5, 56.9, 31.3, 22.5; IR (film): 2930, 1724, 1681, 1649, 1612, 1554, 1541, 1509 cm^{-1}; HRMS ($m\ z^{-1}$): $[M]^+$ calculated for $C_{24}H_{25}N_3O_3$, 403.1896; found, 403.1864.

1-(3-Carboxypropyl)-6-amino-3-(4-benzyloxyphenyl)-pyridazinium bromide (**GZ-i1**)

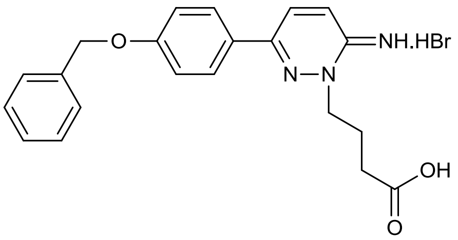

To a solution of $NaHCO_3$ (20 mg, 0.240 mmol), and dimedone (18 mg, 0.128 mmol) in water (0.70 mL) was successively added THF (4.5 mL), triethyl phosphite (11 mg, 0.066 mmol) and palladium(II) acetate (2.0 mg, 0.0089 mmol) under argon. After stirring for 3 min, **6** (51 mg, 0.105 mmol) was added and the mixture was stirred at 35 °C for 17 h. The mixture was diluted with water (5 mL) and washed thoroughly with CH_2Cl_2 (3 × 15 mL). The aqueous phase was separated and evaporated *in vacuo*. The residue was purified by column chromatography ($CHCl_3$:CH_3OH, 9:1 v/v) to give the *acid* **GZ-i1** (26 mg, 0.059 mmol, 56%) as a white solid. m.p.: 180-183 °C; TLC ($CHCl_3$:CH_3OH, 8:2 v/v): RF = 0.30; ^{1}H-NMR ($CDCl_3$:CD_3OD, 1:1 v/v, 600 MHz) δ 8.14 (d, *J* = 12.7 Hz, 1H), 7.88 (d, *J* = 8.8 Hz, 2H), 7.66 (d, *J* = 12.7 Hz, 1H), 7.44 (d, *J* = 7.6 Hz, 2H), 7.39 (dd, *J* = 7.6 and 7.3 Hz, 2H), 7.33 (t, *J* = 7.3 Hz, 1H), 5.15 (s, 2H), 7.11 (d, *J* = 8.8 Hz, 2H), 4.44-4.41 (m, 2H), 2.50-2.45 (m, 2H), 2.19-2.14 (m, 2H); ^{13}C-NMR ($CDCl_3$/CD_3OD 1:1), 150 MHz) δ 177.3, 161.3, 152.2, 150.5, 136.3, 131.1, 128.5, 128.1, 128.0, 127.4, 125.4, 125.0, 115.5, 70.1, 56.2, 30.9, 22.2; IR (film): 2924, 2214, 1712, 1671, 1647, 1609, 1566, 1535, 1512 cm^{-1}; HRMS ($m\ z^{-1}$): $[M]^+$ calculated for $C_{21}H_{22}N_3O_3$, 364.1671; found, 364.1661.

Synthesis of GZ-A1

4-Azidotoluene[2] (**10**)

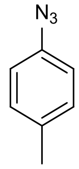

To a solution of 4-toluidine (3.75 g, 35.0 mmol) in HCl (50 mL, 2 M) at –5 °C was added sodium nitrite (2.90 g, 42.0 mmol) in water (10 mL). The temperature was maintained at –5 °C for 5 min. Urea (250 mg, 4.16 mmol) was then added to the reaction mixture. The resulting solution was added over 5 min to a solution of sodium azide (4.55 g, 70.0 mmol) and sodium acetate (8.4 g, 105 mmol) in water (50 mL) at 0 °C. The reaction mixture was stirred at 0 °C for 2 h, then extracted with Et_2O (2 × 100 mL), washed with water (2 × 100 mL), dried ($MgSO_4$) and concentrated *in vacuo*. The residue was dried under high vacuum to give the *azide* **10** (4.00 g, 30.0 mmol, 86%) as a yellow oil. TLC (40-60 °C petroleum ether): RF = 0.25; ^{1}H-NMR ($CDCl_3$, 500 MHz) δ 7.15 (d, *J* = 9.0 Hz, 2H), 6.93 (d, *J* = 9.0 Hz, 2H), 2.33 (s, 3H); ^{13}C-NMR ($CDCl_3$, 125 MHz) δ 137.2, 134.7, 130.4, 118.9, 20.9; IR (film): 2933, 2111 cm^{-1}; HRMS ($m\ z^{-1}$): $[M]^+$ calculated for $C_7H_7N_3$, 133.0640; found, 133.0641.

1-Azido-4-bromomethyl-benzene (**11**)

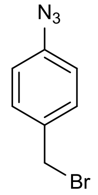

To a solution of **10** (2.27 g, 17.0 mmol) in anhydrous benzene (20 mL) was added NBS (3.78 g, 21.3 mmol) and AIBN (1.40 g, 8.52 mmol). The reaction mixture was heated to 80 °C for 4 h. After cooling to rt, the solvent was concentrated *in vacuo*. The residue was purified by column chromatography (40-60 °C petroleum ether) to give the *azide* **11** (1.60 g, 7.58 mmol, 44%) as a colourless oil. TLC (40-60 °C petroleum ether): RF = 0.30; ^{1}H-NMR ($CDCl_3$, 500 MHz) δ 7.37 (d, *J* = 8.5 Hz, 2H), 7.01 (d, *J* = 8.5 Hz, 2H), 4.48 (s, 2H); ^{13}C-NMR ($CDCl_3$, 125 MHz) δ 140.3, 134.6, 130.9, 119.4, 33.0; IR (film): 2954, 2125 cm^{-1}; HRMS ($m\ z^{-1}$): $[M]^+$ calculated for $C_7H_6N_3{}^{79}Br$, 210.9740, found, 210.9745.

6-[4-(4-Azidobenzyloxy)-phenyl]-pyridazin-3-yl amine (**3**)

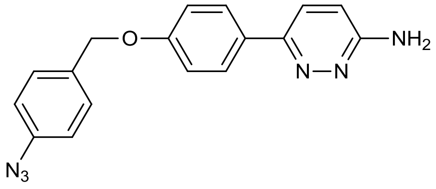

1 (300 mg, 1.60 mmol) and potassium *tert*-butoxide (186 mg, 1.66 mmol) in DMF (2 mL) were cooled to 0 °C. **11** (348 mg, 1.65 mmol) was dissolved in DMF (1 mL) and added dropwise. The reaction mixture was stirred at 0 °C for 3 h. The reaction mixture was extracted with EtOAc (100 mL) and washed with water (100 mL). The aqueous layer was further extracted with EtOAc (4 × 50 mL). The organic extracts were combined and dried ($MgSO_4$). The solvent was removed *in vacuo* and the

residue subjected to column chromatography ($CHCl_3$:CH_3OH, 19:1 v/v) to give the *pyridazine* **3** (193 mg, 0.606 mmol, 38%) as a white solid. m.p.: 204-206 °C; TLC ($CHCl_3$:CH_3OH, 19:1 v/v): RF = 0.20; ^{1}H-NMR (DMSO-d6, 500 MHz) δ 7.88(d, *J* = 8.6 Hz, 2H), 7.74 (d, *J* = 9.6 Hz, 1H), 7.49 (d, *J* = 8.7 Hz, 2H), 7.15 (d, *J* = 8.7 Hz, 2H), 7.07 (d, *J* = 8.6 Hz, 2H), 6.82 (d, *J* = 9.6 Hz, 1H), 6.37 (s, 2H), 5.13 (s, 2H); ^{13}C-NMR (DMSO, 125 MHz) δ 159.4, 158.5, 149.6, 138.9, 133.9, 129.8, 129.5, 126.6, 125.0, 119.2, 115.0, 114.4, 66.7; IR (film): 3431, 3282, 3113, 2954, 2125, 1647 cm^{-1}; HRMS ($m\ z^{-1}$): $[M]^+$ calculated for $C_{17}H_{14}N_6O$, 319.1307; found, 319.1312.

1-(3-Allyloxycarbonyl-propyl)-6-amino-3-[4-(4-azido-benzyloxy)-phenyl]-pyridazinium bromide (**7**)

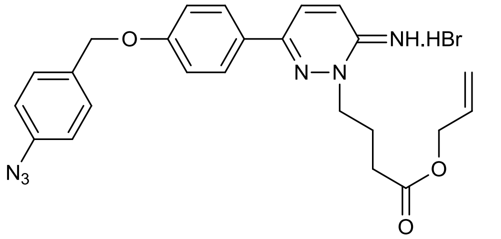

To a solution of **3** (194 mg, 0.609 mmol) in DMF (1 mL) was added allyl-4-bromobutyrate (194 mg, 0.937 mmol). The solution was heated to 80 °C for 6 h. The hot solution was then poured into EtOAc (20 mL) to yield a solid, which was then isolated by filtration. The product was dried under high vacuum to give the *ester* **7** (120 mg, 0.228 mmol, 37%) as a white solid. m.p.: degraded >200 °C; TLC (EtOAc:CH_3OH, 9:1 v/v): RF = 0.20; ^{1}H-NMR (CD_3OD, 500 MHz) δ 8.20 (d, *J* = 9.6 Hz, 1H), 7.88 (d, *J* = 8.6 Hz, 2H), 7.74 (d, *J* = 9.6 Hz, 1H), 7.45 (d, *J* = 8.7 Hz, 2H), 7.07 (d, *J* = 8.6 Hz, 2H), 7.03 (d, *J* = 8.7 Hz, 2H), 5.82 (m, 1H), 5.25 (dd, *J* = 17.3 and 1.1 Hz, 1H), 5.17 (dd, *J* = 10.5 and 1.1 Hz, 1H), 5.11 (s, 2H), 4.48-4.46 (m, 4H), 2.61 (t, *J* = 8.5 Hz, 2H), 2.27 (m, 2H); ^{13}C-NMR (CD_3OD, 125 MHz) δ 174.0, 161.9, 153.8, 152.2, 133.4, 132.5, 131.4, 130.5, 130.5, 129.4, 126.5, 125.0, 120.1, 118.6, 117.0, 70.6, 66.5, 56.8, 31.3, 22.4; IR (film): 3184, 2112, 1727, 1646, 1607, 1543 cm^{-1}; HRMS ($m\ z^{-1}$): $[M]^+$ calculated for $C_{24}H_{25}N_6O_3$, 445.1988; found, 445.1994.

6-Amino-3-[4-(4-azido-benzyloxy)-phenyl]-1-(3-carboxy-propyl)-pyridazinium bromide (**GZ-A1**)

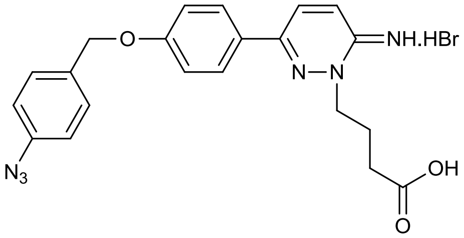

To a solution of **7** (120 mg, 0.228 mmol) in THF (4 mL) and CH_3OH (1 mL) was added morpholine (198 mg, 2.28 mmol) and tetrakis(triphenylphosphine)palladium(0) (26 mg, 0.023 mmol) under argon. The reaction mixture was stirred at rt for 30 min, then concentrated *in vacuo*. The residue was purified by column chromatography ($CHCl_3$:CH_3OH:AcOH, 17:2.9:0.1 v/v) to give the *acid* **GZ-A1** (104 mg, 0.214 mmol, 94%) as a white solid. m.p.: degraded >200 °C; TLC ($CHCl_3$:CH_3OH, 17:3 v/v): RF = 0.20; ^{1}H-NMR ($CDCl_3$:CD_3OD, 1:1 v/v, 600 MHz) δ 8.12 (d, J = 9.5 Hz, 1H), 7.87 (d, J = 8.6 Hz, 2H), 7.71 (d, J = 9.5 Hz, 1H), 7.45 (d, J = 8.4 Hz, 2H), 7.10 (d, J = 8.6 Hz, 2H), 7.06 (d, J = 8.4 Hz, 2H), 5.14 (s, 2H), 4.47-4.41 (m, 2H), 2.37-2.33 (m, 2H), 2.11-2.05 (m, 2H); ^{13}C-NMR ($CDCl_3$:CD_3OD, 1:1 v/v, 150 MHz) δ 177.9, 161.0, 152.2, 150.3, 139.9, 133.1, 131.0, 129.1, 128.7, 128.0, 125.4, 125.3, 119.1, 115.5, 69.4, 56.7, 31.9, 22.7; IR (film): 2930, 2111, 1710, 1656, 1538, 1509 cm^{-1}; UV/vis ($CHCl_3$:CH_3OH, 1:1 v/v): λ_{max} 283 nm (ε = 11900 M^{-1} cm^{-1}), λ_{max} 313 nm (ε = 2200) M^{-1} cm^{-1});; HRMS (m z^{-1}): $[M]^+$ calculated for $C_{21}H_{20}N_3O_3$, 403.1519; found, 403.1529.

Synthesis of GZ-B1

{4-[4-(6-Amino-pyridazin-3-yl)-phenoxymethyl]-phenyl}-phenyl-methanone (**4**)

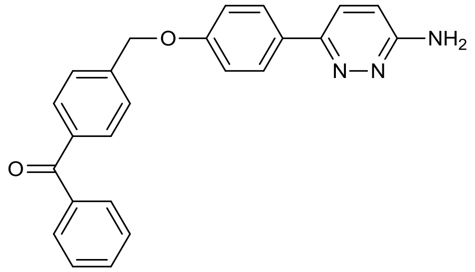

To a solution of potassium *tert*-butoxide (197 mg, 1.76 mmol) in DMF (15 mL) was added a solution of **1** (300 mg, 1.60 mmol) in DMF (15 mL) at 0 °C and the reaction mixture was stirred at 0 °C for 20 min. 4-Bromomethylbenzophenone (485 mg, 1.76 mmol) was added in one portion and the reaction mixture was allowed to warm to rt over 16 h. The reaction mixture was partitioned between sat aq LiCl (50 mL) and EtOAc (200 mL). The aqueous layer was extracted with EtOAc (3 × 100 mL). The organic extracts were combined and dried ($MgSO_4$). The solvent was removed *in vacuo* and the residue subjected to column chromatography (EtOAc) to give the *pyridazine* **4** (266 mg, 0.498 mmol, 44%) as a white solid. m.p.: 167-169 °C; TLC ($CHCl_3$:CH_3OH, 9:1 v/v): RF = 0.30; ^{1}H-NMR (DMSO, 500 MHz) δ 7.90 (d, J = 8.6 Hz, 2H), 7.76 (m, 5H), 7.68 (m, 3H), 7.56 (dd, J = 7.6 and 7.4 Hz, 2H), 7.12 (d, J = 8.6 Hz, 2H), 6.82 (d, J = 9.6 Hz, 1H), 5.29 (s, 2H), 6.38 (s, 2H); ^{13}C-NMR (DMSO, 125 MHz) δ 195.9, 159.9, 158.8, 144.9, 142.5, 137.5, 136.8, 133.2, 130.6, 130.3, 130.0, 129.1, 127.8, 127.1, 125.4, 115.5, 114.8, 69.0; IR (film): 2923, 2491, 1727,

1644, 1596 cm^{-1}; HRMS (*m* z^{-1}): $[M]^+$ calculated for $C_{24}H_{19}N_3O_2$, 381.1472; found, 381.1485.

1-(3-Allyloxycarbonyl-propyl)-6-amino-3-[4-(4-benzoyl-benzyloxy)-phenyl]-pyridazinium bromide (**8**)

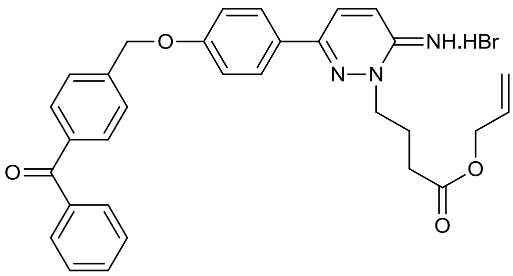

To a solution of **4** (120 mg, 0.31 mmol) in DMF (0.4 mL) was added allyl-4-bromobutyrate (97 mg, 0.47 mmol). The solution was heated to 80 °C for 18 h. The hot solution was then poured into EtOAc (3 mL) to yield a solid, which was then isolated by filtration. The product was dried under high vacuum to give the *ester* **8** (135 mg, 0.229 mmol, 85%) as a white solid. m.p.: 160-162 °C; TLC (EtOAc:CH_3OH, 9:1 v/v): RF = 0.20; ^{1}H-NMR (CD_3OD, 600 MHz) δ 8.31 (d, *J* = 9.6 Hz, 1H), 7.99 (d, *J* = 8.8 Hz, 2H), 7.78 (m, 4H), 7.68-7.66 (m, 3H), 7.63 (d, *J* = 9.6 Hz, 1H), 7.55 (dd, *J* = 7.6 and 7.4 Hz, 2H), 7.20 (d, *J* = 8.8 Hz, 2H), 5.84 (m, 1H), 5.32 (s, 2H), 5.25 (dd, *J* = 17.4 and 1.1 Hz, 1H), 5.17 (dd, *J* = 10.4 and 1.1 Hz, 1H), 4.46-4.48 (m, 4H), 2.64 (t, *J* = 6.8 Hz, 2H), 2.28 (m, 2H); ^{13}C-NMR (CD_3OD, 150 MHz) δ 196.7, 172.6, 161.0, 152.5, 150.4, 141.9, 137.4, 136.9, 132.5, 132.0, 131.2, 130.0, 129.6, 128.2, 128.0, 126.9, 125.5, 125.3, 117.3, 115.3, 69.0, 65.1, 55.5, 29.9, 21.1; IR (film): 3042, 1732, 1644, 1606, 1541 cm^{-1}; HRMS (*m* z^{-1}): $[M]^+$ calculated for $C_{31}H_{30}N_3O_4$, 508.2236; found, 508.2243.

6-Amino-3-[4-(4-benzoyl-benzyloxy)-phenyl]-1-(3-carboxy-propyl)-pyridazinium bromide (**GZ-B1**)

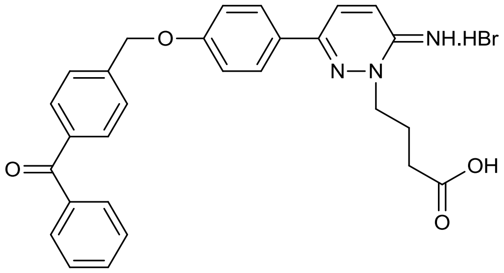

To a solution of **8** (150 mg, 0.254 mmol) in THF (4 mL) and CH_3OH (1 mL) was added 1,4-dimethyl barbituric acid (398 mg, 2.54 mmol) and tetrakis(triphenylphosphine)palladium(0) (29 mg, 0.025 mmol) under argon. The reaction mixture was stirred at rt for 3 h, then concentrated *in vacuo*. The residue

was purified by column chromatography ($CHCl_3$:CH_3OH, 17:3 v/v), then triturated with water to give the *acid* **GZ-B1** (90 mg, 0.164 mmol, 65%) as a white solid. m.p.: 152-154 °C; TLC ($CHCl_3$:CH_3OH, 8:2 v/v): RF = 0.25; ^{1}H-NMR ($CDCl_3$:CD_3OD, 1:1 v/v, 600 MHz) δ 8.13 (d, J = 9.6 Hz, 1H), 7.90 (d, J = 8.2 Hz, 2H), 7.83-7.79 (m, 4H), 7.62-7.59 (m, 4H), 7.49 (dd, J = 7.6 and 7.4 Hz, 2H), 7.14 (d, J = 8.2 Hz, 2H), 5.28 (s, 2H), 4.45-4.41 (m, 2H), 2.42-2.39 (m, 2H), 2.17-2.10 (m, 2H); ^{13}C-NMR ($CDCl_3$:CD_3OD, 1:1 v/v, 150 MHz) δ 198.6, 179.5, 162.3, 153.6, 151.7, 143.0, 138.6, 138.4, 134.2, 132.5, 131.7, 131.3, 129.7, 129.5, 128.4, 126.8, 126.7, 116.9, 70.7, 57.9, 32.8, 23.8; IR (film): 3418, 1734, 1720, 1645, 1578 cm^{-1}; UV/vis (CH_3OH): λ_{max} 278 nm (ε = 12600 M^{-1} cm^{-1}), λ_{max} 326 nm (ε = 1300 M^{-1} cm^{-1}); HRMS (m z^{-1}): [M]$^+$ calculated for $C_{28}H_{26}N_3O_4$, 468.1923; found, 468.1910.

<u>Synthesis of GZ-D1</u>

2,2,2-Trifluoro-1-(4-methylphenyl)-1-ethanone[3] (**12**)

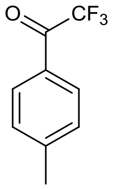

4-Bromotoluene (10.0 g, 58.5 mmol) was dissolved in Et_2O (280 mL) and cooled to –40 °C. *n*-BuLi (40.5 mL, 60.7 mmol, 1.1 M in hexanes) was added dropwise, and the solution was warmed to 0 °C over 2 h. The solution was then cooled to –78 °C and a solution of ethyl trifluoroacetate (9.55 g, 67.2 mmol) in Et_2O (60 mL) was added. The reaction mixture was stirred at –78 °C for 3 h, before being warmed to rt. The solution was hydrolysed with saturated ammonium chloride solution (50 mL), then washed with water (3 × 50 mL) and dried ($MgSO_4$). The solvent was removed *in vacuo* and the residue purified by column chromatography (40-60 °C petroleum ether) to give the *ketone* **12** (4.24 g, 22.5 mmol, 38%) as a colourless oil. TLC (40-60 °C petroleum ether): RF = 0.50; ^{1}H-NMR ($CDCl_3$, 600 MHz) δ 7.97 (d, J = 8.2 Hz, 2H), 7.30 (d, J = 8.2 Hz, 2H), 2.15 (s, 3H); ^{13}C-NMR ($CDCl_3$, 150 MHz) δ 174.9 (q, $^2J_{CF}$ = 37.6 Hz, C=O), 147.4, 130.7, 130.0, 127.3, 118.1 (q, $^1J_{CF}$ = 288.5 Hz, CF_3), 22.8; IR (film): 3433, 1719 cm^{-1}; HRMS (m z^{-1}): [M]$^+$ calculated for $C_9H_7F_3O$, 188.1465; found, 188.1512.

2,2,2-Trifluoro-1-(4-methylphenyl)-1-ethanone oxime[4] (**13**)

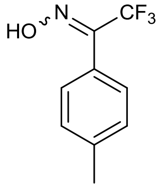

To a solution of **12** (13.5 g, 71.7 mmol) dissolved in pyridine (155 mL), was added hydroxylamine hydrochloride (14.9 g, 215 mmol). The reaction mixture was then heated at 70 °C for 3 h. After cooling to rt, the solvent was removed *in vacuo*. The remaining residue was dissolved in Et_2O (300 mL) and washed with aqueous HCl (300 mL, 0.01 M), water (3 × 50 mL) and dried ($MgSO_4$). The solvent was removed *in vacuo* to give the *oxime* **13** (14.5 g, 71.4 mmol, 99%) as a pale yellow solid used without further purification as a 1:1 mixture of isomers. TLC (40-60 °C petroleum ether:EtOAc, 3:1 v/v): RF = 0.60; ^{1}H-NMR ($CDCl_3$, 600 MHz) δ 8.42 (s, 1H), 8.30 (s, 1H), 7.35-7.25 (m, 8H), 2.43 (s, 6H); ^{13}C-NMR ($CDCl_3$, 150 MHz) δ 148.8 (q, $^2J_{CF}$ = 37.6 Hz, CNO), 141.7, 130.0, 129.0, 123.3, 118.2 (q, $^1J_{CF}$ = 277.5 Hz, CF_3), 21.8; IR (film): 3290, 1888, 1631 cm^{-1}; HRMS (*m* z^{-1}): $[M]^+$ calculated for $C_9H_8F_3NO$, 203.0558; found, 203.0551.

2,2,2-Trifluoro-1-(4-methylphenyl)-1-ethanone *O*-(*p*-toluenesulfonyl) oxime (**14**)

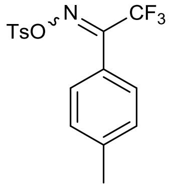

To a solution of **13** (14.5 g, 71.4 mmol) dissolved in pyridine (250 mL) was added *p*-toluenesulfonyl chloride (20.5 g, 107 mmol). The reaction mixture was refluxed at 110 °C for 18 h. After cooling to rt, the solvent was removed *in vacuo*, and the residue was purified by column chromatography (CH_2Cl_2) to give the *tosylate* **14** (19.2 g, 53.5 mmol, 75%) as a white solid used without further purification as a 1:1 mixture of isomers. TLC ($CHCl_3$): RF = 0.80; ^{1}H-NMR ($CDCl_3$, 600 MHz) δ 7.88 (d, *J* = 7.1 Hz, 2H), 7.38 (d, *J* = 7.1 Hz, 2H), 7.34-7.29 (m, 4H), 2.49 (s, 3H), 2.40 (s, 3H); ^{13}C-NMR ($CDCl_3$, 150 MHz) δ 155.0 (q, $^2J_{CF}$ = 36.5 Hz, CNO), 147.5, 132.2, 131.0, 130.3, 130.2, 129.4, 126.8, 123.6, 119.3 (q, $^1J_{CF}$ = 264 Hz, CF_3), 22.0, 21.8; IR (film): 2994, 1917, 1637, 1588, 1489 cm^{-1}; HRMS (*m* z^{-1}): $[M]^+$ calculated for $C_{16}H_{14}F_3NO_3S$, 357.3475; found, 357.3481.

3-*p*-Tolyl-3-trifluoromethyl-diaziridine (**15**)

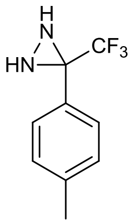

14 (19.2g, 53.6 mmol) was added to a sealed vessel containing Et_2O (130 mL) at –78 °C. Ammonia (25 mL) was condensed in dropwise and the solution was stirred at –78 °C for 8 h. The vessel was then unsealed and allowed to warm to rt. The solution was then extracted with Et_2O (300 mL) and washed with water (300 mL). The organic layer was dried ($MgSO_4$), and the solvent removed *in vacuo*. The product was purified by column chromatography ($CHCl_3$) to give the *diaziridine* **15** (9.46 g, 47.3 mmol, 88%) as a white solid. m.p.: 59-61 °C; TLC ($CHCl_3$): RF = 0.40; ^{1}H-NMR ($CDCl_3$, 600 MHz) δ 7.81 (d, *J* = 8.3 Hz, 2H), 7.36 (d, *J* = 8.3 Hz, 2H), 2.80 (s, 1H), 2.40 (s, 3H), 2.18 (s, 1H); ^{13}C-NMR ($CDCl_3$, 150 MHz) δ 140.0, 138.4, 131.4, 129.9, 123.0 (q, $^1J_{CF}$ = 276.6 Hz, CF_3), 57.7 (q, $^2J_{CF}$ = 30.2 Hz, $C(NH)_2$), 21.8; IR (film): 3005, 1650, 1598, 1489 cm^{-1}; HRMS ($m\ z^{-1}$): $[M]^+$ calculated for $C_9H_9F_3N_2$, 202.1764; found, 202.1753.

3-*p*-Tolyl-3-trifluoromethyl-3*H*-diazirine (**16**)

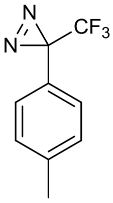

To a solution of **15** (1.00 g, 4.95 mmol) dissolved in CH_2Cl_2 (20 mL) was added triethylamine (2.06 mL, 14.8 mmol) at 0 °C. Iodine (1.38 g, 5.45 mmol) was added gradually, until the solution became brown in colour. The reaction mixture was washed with aqueous NaOH (20 mL, 1 M), water (20 mL), brine (20 mL) and dried ($MgSO_4$). The solvent was carefully removed *in vacuo* at 20 °C owing to the volatility of the product. The residue was purified by column chromatography (40-60 °C petroleum ether:CH_2Cl_2, 20:1 v/v) to give the *diazirine* **16** (601 mg, 3.00 mmol, 61%) as a colourless oil. TLC ($CHCl_3$): RF = 0.90; ^{1}H-NMR ($CDCl_3$, 400 MHz) δ 7.21 (d, *J* = 8.0 Hz, 2H), 7.10 (d, *J* = 8.0 Hz, 2H), 2.39 (s, 3H); ^{13}C-NMR ($CDCl_3$, 150 MHz) δ 140.0, 129.6, 126.5, 126.2, 122.7 (q, $^1J_{CF}$ = 273.0 Hz, CF_3), 28.4 (q, $^2J_{CF}$ = 40.5 Hz, $C(N)_2$), 21.3; IR (film): 3196, 1650 cm^{-1}; HRMS ($m\ z^{-1}$): $[M]^+$ calculated for $C_9H_7F_3N_2$, 200.1065; found, 200.1110.

3-(4-Bromomethylphenyl)-3-trifluoromethyl-3*H*-diazirine (**17**)

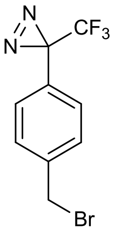

To a solution of **16** (1.80 g, 8.97 mmol) in CCl_4 (40 mL) was added NBS (2.39 g, 13.5 mmol) and AIBN (20 mg, 0.128 mmol). The reaction mixture was refluxed at 70 °C for 4 h. After cooling to rt, the precipitate was filtered and the solvent was removed *in vacuo* at 20 °C owing to the volatility of the product. The residue was purified by column chromatography (40-60 °C petroleum ether: CH_2Cl_2, 20:1 v/v) to give the *bromide* **17** (1.63 g, 5.83 mmol, 65%) as a colourless oil. TLC (40-60 °C petroleum ether:CH_2Cl_2, 19:1 v/v): RF = 0.45; ^{1}H-NMR ($CDCl_3$, 400 MHz) δ 7.46 (d, J = 8.0 Hz, 2H), 7.19 (d, J = 8.0 Hz, 2H), 4.49 (s, 2H); ^{13}C-NMR ($CDCl_3$, 150 MHz) δ 139.5, 129.6, 129.3, 127.0, 122.1 (q, $^1J_{CF}$ = 273.0 Hz, CF_3), 32.1, 28.4 (q, $^2J_{CF}$ = 40.5 Hz, $C(N)_2$); IR (film): 3277, 1644 cm^{-1}; HRMS (m z^{-1}): $[M]^+$ calculated for $C_9H_6{}^{79}BrF_3N_2$, 277.9666; found, 277.9732.

6-{4-[4-(3-Trifluoromethyl-3*H*-diazirin-3-yl)-benzyloxy]-phenyl}-pyridazin-3-ylamine (**5**)

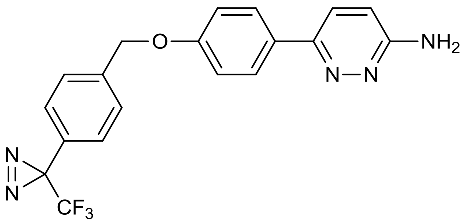

A solution of **1** (160 mg, 0.854 mmol), 18-crown-6 (226 mg, 0.854 mmol) and potassium *tert*-butoxide (96 mg, 0.854 mmol) in DMF (3 mL) were cooled to 0 °C. **17** (140 mg, 0.501 mmol) was dissolved in DMF (1 mL) and added dropwise, then stirred at 0 °C for 3 h. The reaction mixture was extracted with EtOAc (100 mL) and washed with water (100 mL). The aqueous layer was further extracted with EtOAc (4 × 50 mL). The organic extracts were combined and dried ($MgSO_4$). The solvent was removed *in vacuo* and the residue subjected to column chromatography ($CHCl_3$:CH_3OH, 19:1 v/v) to give the *pyridazine* **5** (120 mg, 0.311 mmol, 62%) as a white solid. m.p.: degraded >150 °C; TLC ($CHCl_3$:CH_3OH, 9:1 v/v): RF = 0.25; ^{1}H-NMR (CD_3OD, 500 MHz) δ 7.79 (d, J = 7.7 Hz, 2H), 7.70 (d, J = 9.3 Hz, 1H), 7.55 (d, J = 8.5 Hz, 2H), 7.25 (d, J = 8.5 Hz, 2H), 7.07 (d, J = 7.7 Hz, 2H), 6.99 (d, J = 9.3 Hz, 1H), 5.18 (s, 2H); ^{13}C-NMR (CD_3OD, 150 MHz) δ 160.6, 152.8, 140.6, 133.1, 131.0, 129.7, 129.0, 128.7, 127.8, 127.6, 123.7 (q, $^1J_{CF}$ = 274.7 Hz, CF_3), 117.8, 116.3,

70.2, 29.6 (q, $^2J_{CF}$ = 39.2 Hz, $C(N)_2$); IR (film): 2930, 1654, 1562 cm^{-1}; HRMS (*m* z^{-1}): $[M]^+$ calculated for $C_{19}H_{14}N_5OF_3$, 386.1229; found, 386.1222.

1-(3-Allyloxycarbonyl-propyl)-6-amino-3-{4-[4-(3-trifluoromethyl-3*H*-diazirin-3-yl)-benzyloxy]-phenyl}-pyridazinium bromide (**9**)

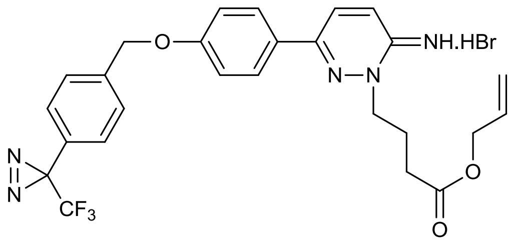

To a solution of **5** (120 mg, 0.311 mmol) in DMF (1 mL) was added allyl-4-bromobutyrate (96 mg, 0.467 mmol). The solution was heated to 80 °C for 5 h. The hot solution was then poured into EtOAc (20 mL) to yield a solid, which was then isolated by filtration. The product was dried under high vacuum to give the *ester* **9** (80 mg, 0.135 mmol, 44%) as a white solid. m.p.: degraded >200 °C; TLC (EtOAc:CH_3OH, 8:2 v/v): RF = 0.20; ^{1}H-NMR (CD_3OD, 600 MHz) δ 8.29 (d, *J* = 9.5 Hz, 1H), 7.94 (d, *J* = 8.9 Hz, 2H), 7.61 (d, *J* = 9.5 Hz, 1H), 7.58 (d, *J* = 8.4 Hz, 2H), 7.28 (d, *J* = 8.4 Hz, 2H), 7.15 (d, *J* = 8.9 Hz, 2H), 5.82 (m, 1H), 5.23-5.22 (m, 3H), 5.15 (dd, *J* = 9.9 and 1.6 Hz, 1H), 4.47-4.43 (m, 4H), 2.62 (t, *J* = 6.7 Hz, 2H), 2.27 (quint, 2H); ^{13}C-NMR (CD_3OD, 150 MHz) δ 174.0, 162.4, 153.9, 151.8, 140.6, 133.4, 132.6, 129.5, 129.1, 128.6, 127.9, 126.9, 126.7, 123.6 (q, $^1J_{CF}$ = 273.9 Hz, CF_3), 118.6, 116.7, 70.2, 66.5, 56.9, 31.3, 29.4 (q, $^2J_{CF}$ = 40.1 Hz, $C(N)_2$), 22.5; IR (film): 3033, 1732, 1647, 1541 cm^{-1}; HRMS (*m* z^{-1}): $[M]^+$ calculated for $C_{26}H_{25}N_5O_3F_3$, 512.1900; found, 512.1900.

6-Amino-1-(3-carboxy-propyl)-3-{4-[4-(3-trifluoromethyl-3*H*-diazirin-3-yl)-benzyloxy]-phenyl}-pyridazinium bromide (**GZ-D1**)

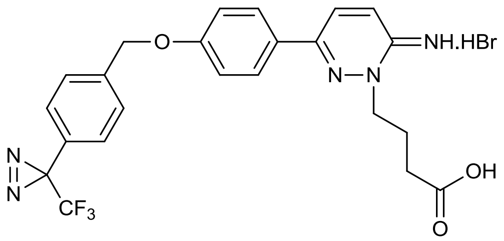

To a solution of **9** (10.0 mg, 0.0168 mmol) in THF (0.2 mL) and CH_3OH (0.05 mL) was added 1,4-dimethyl barbituric acid (26 mg, 0.168 mmol) and tetrakis(triphenylphosphine)palladium(0) (2.0 mg, 0.0017 mmol) under argon. The reaction mixture was stirred at rt for 3 h, then concentrated *in vacuo*. The residue was purified by column chromatography ($CHCl_3$:CH_3OH, 17:3 v/v), then triturated with water to give the *acid* **GZ-D1** (5.5 mg, 0.010 mmol, 59%) as a white solid. m.p.: degraded >200 °C; TLC ($CHCl_3$:CH_3OH, 8:2 v/v): RF = 0.25; ^{1}H-NMR

($CDCl_3$:CD_3OD, 1:1 v/v, 400 MHz) δ 8.16 (d, *J* = 9.6 Hz, 1H), 7.89 (d, *J* = 8.8 Hz, 2H), 7.58 (d, *J* = 9.6 Hz, 1H), 7.53 (d, *J* = 8.0 Hz, 2H), 7.24 (d, *J* = 8.0 Hz, 2H), 7.11 (d, *J* = 8.8 Hz, 2H), 5.20 (s, 2H), 4.43-4.41 (m, 2H), 2.45-2.39 (m, 2H), 2.17-2.10 (m, 2H); ^{13}C-NMR ($CDCl_3$:CD_3OD, 1:1 v/v, 150 MHz) δ 179.5, 162.3, 153.7, 151.8, 140.0, 132.5, 130.0, 129.4, 129.1, 128.0, 126.8, 126.7, 123.5 (q, $^1J_{CF}$ = 272.6 Hz, CF_3), 116.8, 70.4, 57.8, 32.7, 29.4 (q, $^2J_{CF}$ = 40.4 Hz, C(N)$_2$), 23.7; IR (film): 3420, 1719, 1650, 1577 cm^{-1}; UV/vis ($CHCl_3$:CH_3OH, 1:1 v/v) λ_{max} 280 nm (ε = 6100 M^{-1} cm^{-1}), λ_{max} 335 nm (ε = 2100 M^{-1} cm^{-1}); HRMS (*m z^{-1}*): [M]$^+$ calculated for $C_{23}H_{21}N_5O_3F_3$, 472.1596; found, 472.1574

Synthesis of GZ-B2

[4-(6-Amino-pyridazin-3-yl)-phenyl]-phenyl-methanone (**18**)

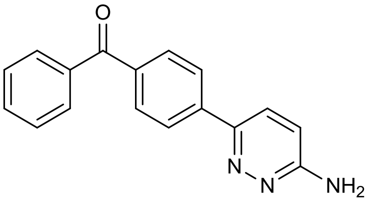

To a microwave vial containing 3-amino-6-chloropyridazine (101 mg, 0.779 mmol), 4-benzoylphenylboronic acid (262 mg, 1.16 mmol), bis(triphenylphosphine)palladium(II) dichloride (27 mg, 0.040 mmol) and K_2CO_3 (201 mg, 1.45 mmol) were added CH_3CN (2.0 mL) and H_2O (1.3 mL). The resulting solution was degassed for 5 min and subjected to microwave irradiation for 10 min at 120 °C. The mixture was diluted with water (50 mL), extracted with EtOAc (3 × 100 mL), washed with brine (100 mL) and dried ($MgSO_4$). The solvent was concentrated *in vacuo*. The residue was purified by column chromatography (EtOAc:CH_3OH, 19:1 v/v) to give the *pyridazine* **18** (130 mg, 0.472 mmol, 61%) as a white solid. m.p.: 147-149 °C; TLC (EtOAc): RF = 0.15; ^{1}H-NMR (CD_3OD, 500 MHz) δ 8.00 (s, *J* = 7.5 Hz, 2H), 7.85 (d, *J* = 7.9 Hz, 2H), 7.77 (m, 3H), 7.61 (t, *J* = 7.5 Hz, 1H), 7.49 (dd, *J* = 7.9 and 7.5 Hz, 2H), 7.02 (d, *J* = 9.3 Hz, 1H); ^{13}C-NMR (CD_3OD, 125 MHz) δ 198.1, 160.4, 151.4, 141.5, 138.4, 138.3, 133.7, 131.6, 130.9, 129.3, 128.3, 126.8, 117.4; IR (film): 3052, 2923, 1727, 1644, 1596 cm^{-1}; HRMS (*m z^{-1}*): [M]$^+$ calculated for $C_{17}H_{14}N_3O$, 276.1137; found, 276.1141.

1-(3-Allyloxycarbonyl-propyl)-6-amino-3-(4-benzoyl-phenyl)-pyridazinium bromide (**19**)

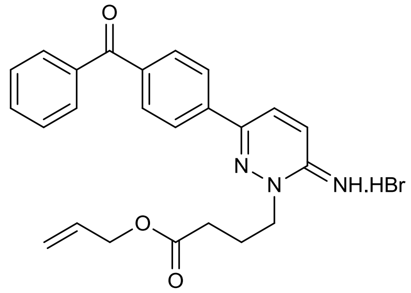

To a solution of **18** (100 mg, 0.363 mmol) in DMF (1 mL) was added allyl-4-bromobutyrate (103 mg, 0.497 mmol). The solution was heated to 80 °C for 18 h. The hot solution was then poured into EtOAc (20 mL) to yield a solid, which was then isolated by filtration. The product was dried under high vacuum to give the *ester* **19** (133 mg, 0.276 mmol, 76%) as a white solid. m.p.: 142-144 °C; TLC (EtOAc:CH_3OH, 8:2 v/v): RF = 0.20; ^{1}H-NMR ($CDCl_3$:CD_3OD, 1:1 v/v, 600 MHz) δ 8.45 (d, *J* = 9.5 Hz, 1H), 8.18 (d, *J* = 8.2 Hz, 2H), 7.95 (d, *J* = 7.8 Hz, 2H), 7.82 (d, *J* = 8.2 Hz, 2H), 7.74 (d, *J* = 9.5 Hz, 1H), 7.71 (t, *J* = 7.6 Hz, 1H), 7.57 (dd, *J* = 7.8 and 7.6 Hz, 2H), 5.86 (m, 1H), 5.27 (dd, *J* = 17.2 and 1.2 Hz, 1H), 5.17 (dd, *J* = 10.4 and 1.2 Hz, 1H), 4.54-4.51 (m, 4H), 2.67 (t, *J* = 6.7 Hz, 2H), 2.32 (quint, 2H); ^{13}C-NMR (CD_3OD, 150 MHz) δ 196.1, 172.7, 153.1, 149.7, 139.3, 136.9, 136.4, 132.9, 132.0, 131.5, 130.3, 129.7, 128.3, 126.4, 125.7, 117.2, 65.1, 55.7, 29.9, 21.1; IR (film): 3165, 2999, 1725, 1648, 1533 cm^{-1}; HRMS ($m\ z^{-1}$): $[M]^+$ calculated for $C_{24}H_{24}N_3O_3$, 402.1818; found, 402.1822.

6-Amino-3-(4-benzoyl-phenyl)-1-(3-carboxy-propyl)-pyridazinium bromide (**GZ-B2**)

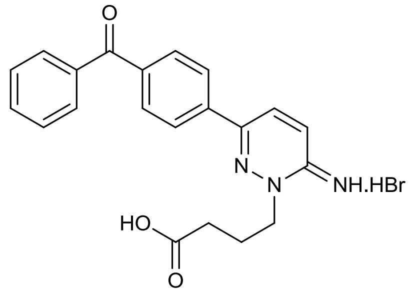

To a solution of **19** (64 mg, 0.133 mmol) in THF (4 mL) and CH_3OH (1 mL) was added morpholine (116 mg, 1.33 mmol) and tetrakis(triphenylphosphine)palladium(0) (15 mg, 0.0133 mmol) under argon. The reaction mixture was stirred at rt for 30 min, then concentrated *in vacuo*. The residue was purified by column chromatography ($CHCl_3$:CH_3OH:AcOH, 17:2.9:0.1 v/v) to give the *acid* **GZ-B2** (54 mg, 0.122 mmol, 92%) as a white solid. m.p.: 140-142 °C; TLC ($CHCl_3$:CH_3OH, 8:2 v/v): RF = 0.25; ^{1}H-NMR ($CDCl_3$:CD_3OD, 1:1 v/v, 600 MHz) δ 8.25 (d, *J* = 9.5 Hz, 1H), 8.09 (d, *J* = 8.2 Hz, 2H), 7.93 (d, *J* = 7.7 Hz, 2H), 7.80 (d, *J* = 8.2 Hz, 2H), 7.78 (d, *J* = 9.5 Hz,

1H), 7.67 (t, J = 7.5 Hz, 1H), 7.54 (dd, J = 7.7 and 7.5 Hz, 2H), 4.51-4.47 (m, 2H), 2.43-2.37 (m, 2H), 2.15-2.10 (m, 2H); ^{13}C-NMR ($CDCl_3$:CD_3OD, 1:1 v/v, 150 MHz) δ 196.6, 179.3, 152.9, 149.3, 139.3, 136.8, 136.3, 133.1, 131.1, 130.7, 130.0, 128.5, 126.4, 126.1, 56.9, 31.9, 22.7; IR (film): 2933, 1740, 1651, 1570, 1533, 1511 cm^{-1}; UV/vis ($CHCl_3$:CH_3OH, 1:1 v/v): λ_{max} 306 nm (ε = 10600 M^{-1} cm^{-1}); HRMS (*m z*$^{-1}$): $[M]^+$ calculated for $C_{21}H_{20}N_3O_3$, 362.1505; found, 362.1510.

<u>Synthesis of GZ-B1-biotin</u>

(3-iodo-4-methylphenyl)(phenyl)methanone (**20**)

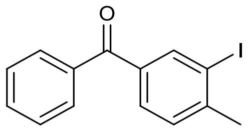

To neat 2-iodo-4-methylbenzoic acid (5.00 g, 19.1 mmol) was added thionyl chloride (7.00 mL, 96.5 mmol) and the reaction mixture was heated to reflux for 2 h. The reaction mixture was allowed to cool and then was concentrated *in vacuo*. The residue was redissolved in benzene (20 mL) and $AlCl_3$ (2.80g, 21.0 mmol) was added. The mixture was then heated to 50°C for 3 h. After having cooled, the reaction mixture was partitioned between 3M aq HCl (20 mL), and EtOAc (20 mL). The aqueous layers were extracted with EtOAc (3 × 20 mL), and the combined organic layers were washed with brine (50 mL), dried ($MgSO_4$) and concentrated *in vacuo*. The residue was purified by column chromatography (40-60 °C petroleum ether:Et_2O, 9:1 v/v) to give **20** (5.09 g, 15.8 mmol, 83%) as a white solid. m.p.: 78-79 °C; ^{1}H-NMR ($CDCl_3$, 500 MHz) δ 8.25 (d, J = 1.6 Hz, 1H), 7.76-7.79 (m, 2H), 7.68 (dd, J = 7.9, 1.7 Hz, 1H), 7.60 (tt, J = 7.3, 1.3 Hz, 1H) 7.49 (dd, J = 7.9, 7.6 Hz, 2H), 7.34 (d, J = 7.9 Hz, 1H), 2.52 (s, 3H); ^{13}C-NMR ($CDCl_3$, 125 MHz) δ 194.9, 146.4, 140.5, 137.3, 136.9, 132.7, 130.0, 130.0, 129.5, 128.5, 100.8, 28.5; IR (solid) 3050, 1722, 1652, 1595, 1587, 1577, 1544 cm^{-1}; HRMS ($[M+H]^+$) calculated for $C_{14}H_{11}IO$ 321.98491; found 321.98516.

(4-(bromomethyl)-3-iodophenyl)(phenyl)methanone (**21**)

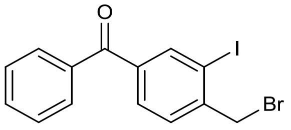

To a solution of **20** (955 mg, 2.96 mmol) in benzene (20 mL) were added NBS (527 mg, 2.96 mmol) and AIBN (63 mg, 0.39 mmol). The reaction mixture was heated to 80 °C for 16 h then the reaction solvent was removed *in vacuo*. The residue was purified by column chromatography (40-60 °C petroleum ether: Et_2O, 100:1 v/v) to

give starting material **20** (372 mg, 39%). Further elution gave **21** (666 mg, 1,66 mmol, 56%) as a white solid. m.p.: 54-56 °C; ^{1}H-NMR ($CDCl_3$, 500 MHz) δ 8.27 (d, *J* = 1.9 Hz, 1H), 7.79 (dt, *J* = 7.6, 1.9 Hz, 2H), 7.74 (dd, *J* = 7.9, 1.9 Hz, 1H), 7.62 (tt, *J* = 7.6, 1.9 Hz, 1H), 7.57 (d, *J* =7.9 Hz, 1H), 7.50 (t, *J* = 7.6 Hz, 2H), 4.63 (s, 2H); ^{13}C-NMR ($CDCl_3$, 125 MHz) δ 194.4, 144.3, 141.3, 140.0, 136.8, 133.1, 130.4, 130.2, 130.1, 128.6, 99.7, 37.8; IR (solid) 3030, 2927, 1734, 1649, 1594, 1562, 1550, 1510 cm^{-1}; HRMS $[^{79}M]^+$ calculated for $C_{14}H_{10}BrIO$ 399.89542; found 399.89584.

(4-((4′-(6″-aminopyridazin-3″-yl)phenoxy)methyl)-3-iodophenyl)(phenyl)methanone (**22**)

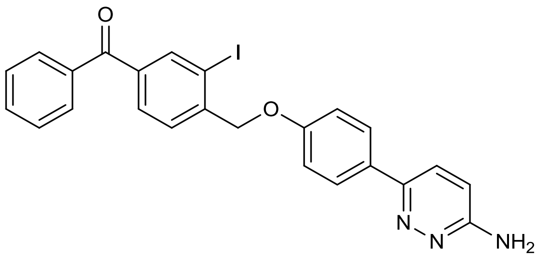

To a solution of NaH (131 mg, 3.56 mmol) in DMF (5 mL) at 0 °C was **1** (606 mg, 3.24 mmol) in one portion. The resulting solution was stirred at 0 °C for 20 min before a solution of **21** (1.56 g, 3.88 mmol) in DMF (5 mL) was added via cannula. The reaction mixture was the stirred at rt for 4 h, quenched with H_2O (2 mL), and concentrated *in vacuo*. The residue was purified by column chromatography (EtOAc) to give **22** (1.25g, 2.47 mmol, 82%) as a white solid. m.p: 90-91 °C; ^{1}H-NMR ($CDCl_3$, 600 MHz) δ 8.31 (d, *J* = 1.5Hz, 1H), 7.94 (d, *J* = 8.9 Hz, 2H), 7.77-7.81 (m, 3H), 7.65 (d, *J* = 8.0 Hz, *1H*), 7.59-7.63 (m, 2H), 7.50 (t, *J* = 7.7 Hz, 2H), 7.09 (d, *J* = 8.9 Hz, 2H), 6.84 (d, *J* = 8.9 Hz, 1H), 5.16 (s, 2H), 4.82 (br s, 2H); ^{13}C-NMR ($CDCl_3$, 125 MHz) δ 194.9, 159.0, 158.1, 153.3, 143.4, 140.5, 138.6, 137.0, 133.0, 130.1, 130.2, 130.1, 128.6, 128.0, 128.0, 127.7, 125.9, 115.3, 115.3, 115.1, 96.4, 73.8; IR (solid) 3420, 3312, 3168, 2928, 2349, 1653 cm^{-1};HRMS $[M]^+$ calculated for $C_{24}H_{18}IN_2O_2$ 507.04382; found 507.04450.

6-(2-((4-(6-aminopyridazin-3-yl)phenoxy)methyl)-5-benzoylphenyl)hex-5-ynoic acid (**23**)

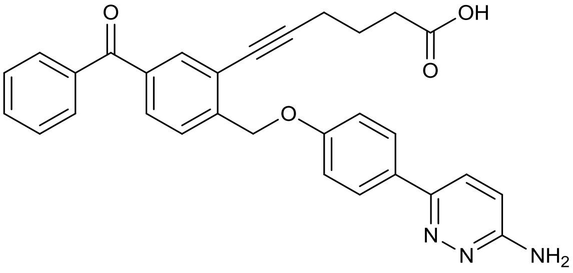

To a solution of **22** (160 mg, 0.32 mmol) in degassed Et_3N (5 mL) and THF (5 mL) was added 5-hexynoic acid (38 µL, 0.35 mmol), $Pd(PPh_3)_3$ (7.3 mg, 0.006 mmol) and CuI (1.8 mg, 0.010 mmol). The reaction mixture was heated to 65 °C for 16 h in the dark. Methanol (20 mL) was added and the reaction mixture was concentrated *in vacuo*. The residue was purified by column chromatography ($CHCl_3$: MeOH, 10:1 v/v) to give **23** (76.6 mg, 49%) as an insoluble white solid. m.p. 186-188 °C; R_f = 0.30 ($CHCl_3$/CH_3OH 9:1); IR (film) 3328, 1740, 1638 cm^{-1}; ^{1}H-NMR and ^{13}C-NMR were not obtained due to high insolubility of compound. Purity was established by chemical derivation. HRMS (*m* z^{-1}): $[M]^+$ calculated for $C_{30}H_{26}N_3O$ 492.1923; found 492.1901.

tert butyl *N*-{2-[2′-(2″-aminoethoxy)ethoxy]ethyl}carbamate (**26**)

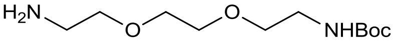

To a solution of 2-[2-(2-aminoethoxy)ethoxy]ethanamine (8.00 g, 54.0 mmol) in CH_2Cl_2 (20 mL) at 0 °C, was added a solution of Boc_2O (1.18 g, 5.40 mmol) in CH_2Cl_2 (20 mL) dropwise. The reaction mixture was stirred at rt for 16 h before being concentrated *in vacuo*. The resulting reside was redissolved in CH_2Cl_2 (40 mL), washed with H_2O (40 mL). The aqueous layer was extracted with CH_2Cl_2 (2×40 mL) and the combined organic layers were dried and concentrated *in vacuo* to give **26** (1.38 g, 5.54 mmol, 99%) as a white solid. m.p.: 234 °C; ^{1}H-NMR ($CDCl_3$, 600 MHz) δ 5.16 (br s, 1H), 3.61 (s, 4H), 3.51-3.56 (m, 4H), 3.32 (q, *J* =5.2 Hz, 2H), 2.89 (t, *J* =5.2 Hz, 2H), 1.87 (br s, 2H), 1.43 (s, 9H); ^{13}C-NMR ($CDCl_3$, 125 MHz) δ 156.1, 79.2, 72.6, 70.3, 70.2, 70.2, 41.4, 40.4, 28.5; IR (solid) 3366, 2973, 2927, 2865, 1703 cm^{-1}; HRMS (CI^+) $[M+H]^+$ calculated for $C_{11}H_{24}N_2O_4$ 249.18088; found 249.17974.

N-(2-(2′-(2″-(*N*-Boc-amino)ethoxy)ethoxy)ethyl)biotinylamine (**27**)

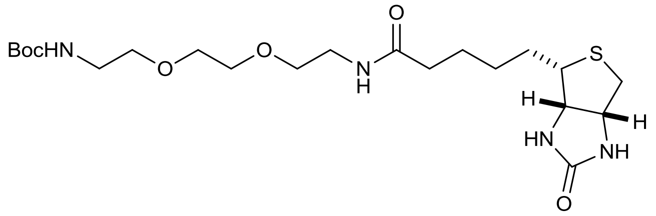

To a solution of D-biotin (221 mg, 0.906 mmol) and HBTU (298 mg, 0.785 mmol) in DMF (3 mL) was added DIPEA (310 µL, 1.81 mmol). The reaction mixture was stirred for 20 min at rt before being added via cannula to a solution of **26** (150 mg, 0.604 mmol) in DMF (5 mL). The reaction mixture was stirred for 2 h before the solvent was removed *in vacuo*. The residue was purified by column chromatography (CH_2Cl_2: CH_3OH, 20:1 v/v) to give **27** (182.5 mg, 0.366 mmol, 63%) as a colourless oil. ^{1}H-NMR (CD_3OD, 600 MHz) δ 4.50 (dd, *J* 7.8, 4.3 Hz, 1H), 4.31 (dd, *J* = 7.8, 4.8 Hz, 1H), 3.61 (app s, 4H), 3.55 (t, *J* = 5.5 Hz, 2H), 3.52 (t, *J* = 5.7 Hz, 2H), 3.37 (t, J = 5.5 Hz, 2H), 3.19-3.24 (m, 3H), 2.93 (dd, 12.7, 4.8 Hz, 1H), 2.71 (d, J = 12.7 Hz, 1H), 2.22 (t, J = 7.2 Hz, 2H), 1.56-1.77 (m, 4H), 1.42-1.48 (m, 11H); ^{13}C-NMR (CD_3OD, 125 MHz) δ 176.2, 166.1, 158.4, 80.1, 71.3, 71.1, 70.6, 63.4, 61.6, 57.0, 41.2, 41.1, 40.3, 36.7, 29.8, 29.5, 28.9, 26.9; IR (oil) 3292, 2930, 2867, 1693 cm^{-1}; HRMS (ES) $[M+Na]^+$ calculated for $C_{21}H_{38}N_4O_6SNa$, 497.2410; found 497.2423.

N-(2-(2′-(2″-aminoethoxy)ethoxy)ethyl)biotinylamine TFA salt (**28**)

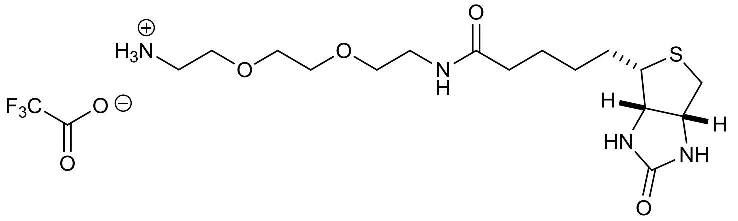

To a solution of **27** (700 mg) in CH_2Cl_2 (4 mL) was added TFA (4 mL). The reaction mixture was stirred at rt for 3 h before being concentrated *in vacuo* to give **28** (725 mg, 100%) as a yellow oil. ^{1}H-NMR (CD_3OD, 600 MHz) δ 4.51 (dd, *J* = 7.9, 4.7 Hz, 1H), 4.32 (dd, *J* =7.9, 4.5 Hz, 1H), 3.70 (t, *J* = 4.8 Hz, 2H), 3.64-3.68 (m, 4H), 3.56 (t, *J* = 5.7 Hz, 2H), 3.37 (t, *J* = 5.7 Hz, 2H), 3.21-3.23 (m, 1H), 3.12 (t, *J* = 4.8 Hz, 2H), 2.93 (d, *J* = 12.8 Hz, 1H), 2.71 (d, *J* = 12.8 Hz, 1H), 2.23 (t, *J* = 7.3 Hz, 2H), 1.55-1.77 (m, 4H), 1.41-1.48 (m, 2H); ^{13}C-NMR (CD_3OD, 150 MHz) δ 176.3, 166.2, 161.3 (q, *J* = 38 Hz) 117.3 (q, *J* = 289 Hz), 71.4, 71.3, 70.7, 63.4, 61.7, 57.0, 41.7, 40.7, 40.2, 36.7, 29.7, 29.5, 26.9; IR (oil) 3293, 3075, 2929, 1777, 1673 cm^{-1}; HRMS (CI^+) $[M+H]^+$ calculated for $C_{16}H_{31}N_4O_4S$, 375.20605; found 375.20509.

6-(2-((4-(6-aminopyridazin-3-yl)phenoxy)methyl)-5-benzoylphenyl)-N-(2-(2-(2-(biotinylamino)ethoxy)ethoxy)ethyl)hex-5-ynamide (**24**)

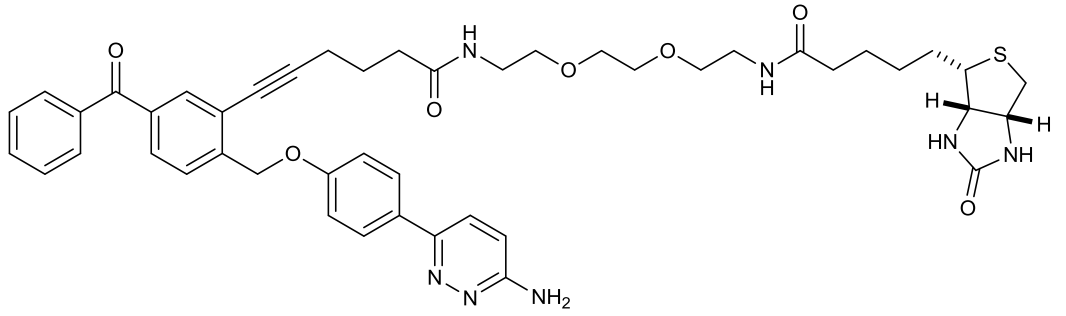

To a solution of **23** (126 mg, 0.26 mmol) in DMF (3 mL) was added DCC (58 mg, 0.28 mmol) and the resulting mixture was stirred at 0 °C for 30 min. The reaction was allowed to warm to rt over 20 min before Et_3N (36 μL, 0.26 mmol) and **28** (125 mg, 0.26 mmol) were added. The reaction mixture was then stirred at rt for 16 h before being diluted with EtOAc (3 mL) and washed with sat aq LiCl, dried ($MgSO_4$) and concentrated *in vacuo*. The residue was purified by column chromatography ($CHCl_3$:CH_3OH, 9:1 v/v) to give **24** (113 mg, 52%) as a yellow oil. ^{1}H-NMR (CD_3OD, 600 MHz) δ 7.91 (s, 1H), 7.87 (d, *J* = 8.6 Hz, 2H), 7.76-7.80 (m, 3H), 7.70-7.75 (m, 2H), 7.67 (t, *J* = 7.4 Hz, 1H), 7.56 (t, *J* = 7.9 Hz, 2H), 7.14 (d, *J* = 8.6 Hz, 2H), 7.01 (d, *J* = 9.4 Hz, 1H), 5.38 (s, 2H), 4.46 (dd, *J* = 8.0, 4.5 Hz, 1H), 4.26 (dd, *J* = 8.0, 4.4 Hz, 1H), 3.57 (app s, 3H), 3.50 (t, *J* = 5.4 Hz, 4H), 3.32-3.35 (m, 4H), 3.13-3.17 (m, 1H), 2.89 (d, *J* = 12.8 Hz, 1H), 2.68 (d, *J* = 12.8 Hz, 1H), 2.53 (t, *J* = 7.6 Hz, 2H), 2.36 (t, *J* = 7.4 Hz, 2H), 2.17 (t, *J* = 7.3 Hz, 2H) 1.89 (quint, *J* = 7.2 Hz, 2H), 1.51-1.73 (m, 4H), 1.37-1.42 (m, 2H); ^{13}C-NMR (CD_3OD, 150 MHz) δ 197.4, 176.1, 175.4, 163.6, 160.9, 144.1, 138.5, 138.4, 134.4, 134.1, 131.2, 131.0, 131.0, 130.4, 129.7, 128.6, 128.0, 124.2, 117.5, 116.2, 97.3, 79.5, 78.7, 71.3, 70.6 70.6, 69.2, 66.9, 63.3, 61.6, 57.0, 41.0, 40.3, 40.3, 36.7, 36.0, 29.8, 29.5, 26.8, 25.9, 19.2; IR (oil) 3301, 2926, 2856, 2349, 1693, 1650 cm^{-1}; HRMS (ES^+) $[M+Na]^+$ calculated for $C_{46}H_{53}N_7O_7SNa$, 870.3625; found 870.3621.

allyl 4-(3-(4-((4-benzoyl-2-(5,16-dioxo-1-((3aS,4S,6aR)-2-oxohexahydro-1H-thieno[3,4-d]imidazol-4-yl)-9,12-dioxa-6,15-diazahenicos-20-yn-21-yl)benzyl)oxy)phenyl)-6-iminopyridazin-1(6H)-yl)butanoate hydrobromide (**25**)

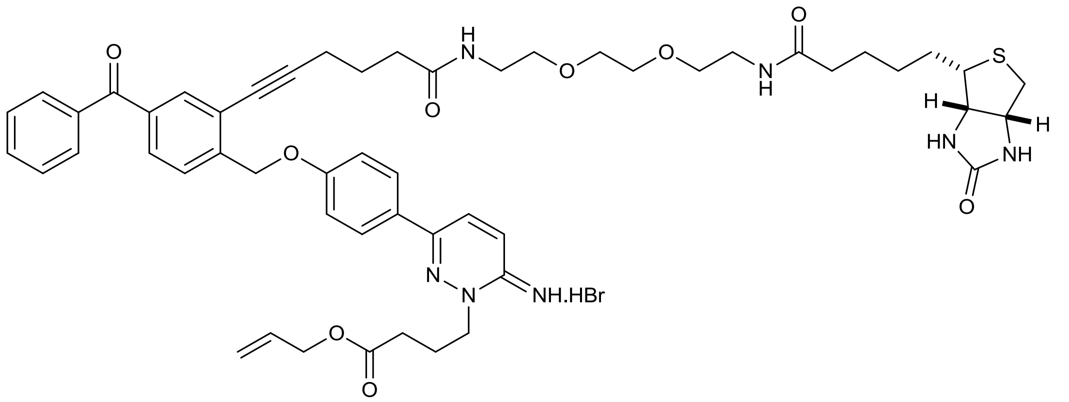

To a solution of **24** (19 mg, 0.02 mmol) in DMF (0.1 mL) was added allyl 4-bromobutyrate (7.0 mg, 0.03 mmol) and heated to 120 °C for 16 h. Cold EtOAc (2 mL) was added and the resulting residue filtered to give **25** (18.2 mg, 77%) as a brown oil. ^{1}H-NMR (CD_3OD, 600 MHz) δ 8.32 (d, *J* = 9.6 Hz, 1H), 7.99 (d, *J* = 8.8 Hz, 2H), 7.78 (s, 1H), 7.72 (td, *J* = 7.9, 1.7 Hz, 1H), 7.70 (d, *J* = 8.1 Hz, 1H), 7.67 (t, *J* = 7.4 Hz, 1H), 7.62 (d, *J* = 9.6 Hz, 1H), 7.56 (t, *J* = 7.8 Hz, 2H), 7.20 (d, *J* = 9.0 Hz, 2H), 5.80-5.88 (m, 1H), 5.41 (s, 2H), 5.24 (dd, *J* = 17.3, 1.3 Hz, 1H), 5.16 (dd, *J* = 10.5, 1.3 Hz, 1H), 4.44-4.50 (m, 5H), 4.28 (dd, *J* = 7.7, 7.5 Hz, 1H), 3.58 (app s, 4H) 3.52 (app q, *J* = 5.8 Hz, 4H), 3.32-3.36 (m, 4H), 3.14-3.20 (m, 1H), 2.90 (dd, *J* = 12.6, 4.9 Hz, 1H), 2.68 (d, *J* = 13.0 Hz, 1H), 2.63 (t, *J* = 6.8 Hz, 2H), 2.53 (t, *J* = 7.0 Hz, 2H), 2.37 (t, *J* = 7.3 Hz, 2H), 2.27 (t, *J* = 6.8 Hz, 2H), 2.18 (t, *J* = 7.3 Hz, 2H), 1.89 (quint, *J* = 7.3 Hz, 2H), 1.50-1.74 (m, 4H), 1.37-1.44 (m, 2H); ^{13}C-NMR (CD_3OD, 150 MHz) δ 197.3, 175.9, 175.4, 174.0, 162.5, 160.5, 158.8, 153.9, 152.9, 144.0, 134.5, 134.1, 133.6, 133.5, 131.0, 130.4, 129.7, 129.5, 127.2, 127.1, 126.7, 122.5, 118.7, 116.7, 97.3, 81.6, 71.3, 70.6, 70.6, 69.3, 66.5, 63.3, 61.6, 56.9, 55.9, 43.8, 41.1, 40.3, 40.3, 36.7, 36.0, 31.3, 29.8, 29.5, 26.9, 25.9, 22.5, 19.8: IR (oil) 3323, 2939, 1726 cm^{-1}; HRMS (ES+) $[M+H]^+$ calculated for $C_{53}H_{64}N_7O_9S$ 974.4486; found 974.4557.

4-(3-(4-((4-benzoyl-2-(5,16-dioxo-1-((3aS,4S,6aR)-2-oxohexahydro-1H-thieno[3,4-d]imidazol-4-yl)-9,12-dioxa-6,15-diazahenicos-20-yn-21-yl)benzyl)oxy)phenyl)-6-iminopyridazin-1(6H)-yl)butanoate hydrochloride (**GZ-B1-biotin**)

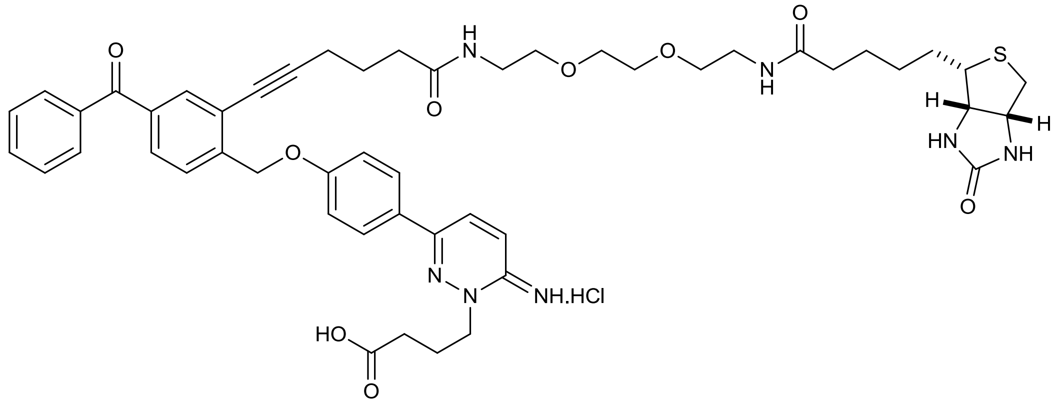

To a solution **25** (8.6 mg) in THF (1 mL) and H_2O (1 mL) was added NaOH (1.0 mg) and the reaction mixture was stirred at 50 °C for 3 h. After cooling to 10 °C the reaction mixture was washed with EtOAc (2 mL) and the aqueous layer separated then acidified to pH 1 by addition of 0.1 M aq HCl. The aqueous layer was stirred at 0 °C for 1 h, then the solvent was removed *in vacuo* and the residue triturated once with H_2O, to give **GZ-B1-biotin** (11.9 mg, 65%) as a brown solid. m.p: degraded >150 °C; ^{1}H-NMR (CD_3OD, 600 MHz) δ 8.30 (d, J = 9.6 Hz, 1H), 7.99 (d, J = 9.0 Hz, 2H), 7.77-7.80 (m, 1H), 1.7 Hz, 1H), 7.72 (dd, J = 8.1, 1.7 Hz, 1H) 7.70 (d, J = 8.1 Hz, 1H), 7.65-7.69 (m, 1H), 7.62 (d, J = 9.6 Hz, 1H), 7.56 (t, J = 7.8 Hz, 2H), 7.20 (d, J = 9.0 Hz, 2H), 5.41 (s, 2H), 4.42-4.48 (m, 3H), 4.25-4.28 (m, 1H), 3.58 (app s, 4H) 3.51 (app q, J = 5.7 Hz, 4H), 3.32-3.36 (m, 4H), 3.14-3.18 (m, 1H), 2.90 (dd, J = 12.7, 4.9 Hz, 1H), 2.68 (d, J = 12.7 Hz, 1H), 2.50-2.54 (m, 4H), 2.36 (t, J = 7.4 Hz, 2H), 2.15-2.24 (m, 4H), 1.88 (quint, J = 7.2 Hz, 2H), 1.52-1.73 (m, 4H), 1.37-1.43 (m, 2H); ^{13}C-NMR (CD_3OD, 150 MHz) δ 197.4, 177.1, 176.1, 175.4, 173.7, 162.5, 153.0, 151.8, 144.0, 138.4, 134.5, 134.1, 132.6, 131.0, 130.4, 129.7, 129.5, 128.7, 127.1, 126.6, 124.5, 116.6, 98.0, 78.6, 71.3, 70.6, 70.6, 69.6, 69.3, 63.3, 61.6, 57.0, 41.0, 40.3, 40.2, 36.7, 36.0, 31.6, 29.8, 29.5, 26.9, 25.9, 23.0, 19.8; IR (oil) 3263, 2832, 2380, 1650 cm^{-1}; UV/vis (CH_3OH): λ_{max} 274 nm (ε = 140 M^{-1}cm^{-1}); HRMS (ESI$^+$) [M+H]$^+$ calculated for $C_{50}H_{59}N_7O_9S$ 934.4173; found 934.4884.

Supplementary References

---

[1] Calculated LogP values from OSIRIS Property Explorer, Organic Chemistry Portal. Available at www.organic-chemistry.org. Accessed March 2014.

[2] Demko, Z.P.; Sharpless, K.B. A click chemistry approach to tetrazoles by Huisgen 1,3-dipolar cycloaddition: synthesis of 5-sulfonyl tetrazoles from azides and sulfonyl cyanides. *Angew. Chem Int. Ed Engl.* **41**, 2110-2113 (2002).

[3] Creary,X. Reaction of organometallic reagents with ethyl trifluoroacetate and diethyl oxalate. Formation of trifluoromethyl ketones and α-keto esters via stable tetrahedral adducts. *J. Org. Chem.* **52**, 5026-5030 (1987).

[4] Strømgaard,K. et al. Ginkgolide derivatives for photolabeling studies: preparation and pharmacological evaluation. *J Med Chem* **45**, 4038-4046 (2002).
